# TP53RK Drives the Progression of Chronic Kidney Disease by Phosphorylating Birc5

**DOI:** 10.1002/advs.202301753

**Published:** 2023-06-29

**Authors:** Mengqiu Wu, Qianqian Jin, Xinyue Xu, Jiaojiao Fan, Weiyi Chen, Mengqiu Miao, Ran Gu, Shengnan Zhang, Yan Guo, Songming Huang, Yue Zhang, Aihua Zhang, Zhanjun Jia

**Affiliations:** ^1^ Department of Nephrology Nanjing Key Laboratory of Pediatrics Jiangsu Key Laboratory of Pediatrics Children's Hospital of Nanjing Medical University Nanjing Medical University Nanjing 210008 P. R. China; ^2^ School of Medicine Southeast University Nanjing 210009 P. R. China; ^3^ Department of Emergency Medicine Children's Hospital of Nanjing Medical University Nanjing 210008 P. R. China

**Keywords:** TP53RK, Birc5, chronic kidney disease, fibrosis, renal tubular cells, renal fibroblasts, phosphorylation

## Abstract

Renal fibrosis is a common characteristic of various chronic kidney diseases (CKDs) driving the loss of renal function. During this pathological process, persistent injury to renal tubular epithelial cells and activation of fibroblasts chiefly determine the extent of renal fibrosis. In this study, the role of tumor protein 53 regulating kinase (TP53RK) in the pathogenesis of renal fibrosis and its underlying mechanisms is investigated. TP53RK is upregulated in fibrotic human and animal kidneys with a positive correlation to kidney dysfunction and fibrotic markers. Interestingly, specific deletion of TP53RK either in renal tubule or in fibroblasts in mice can mitigate renal fibrosis in CKD models. Mechanistic investigations reveal that TP53RK phosphorylates baculoviral IAP repeat containing 5 (Birc5) and facilitates its nuclear translocation; enhanced Birc5 displays a profibrotic effect possibly via activating PI3K/Akt and MAPK pathways. Moreover, pharmacologically inhibiting TP53RK and Birc5 using fusidic acid (an FDA‐approved antibiotic) and YM‐155(currently in clinical phase 2 trials) respectively both ameliorate kidney fibrosis. These findings demonstrate that activated TP53RK/Birc5 signaling in renal tubular cells and fibroblasts alters cellular phenotypes and drives CKD progression. A genetic or pharmacological blockade of this axis serves as a potential strategy for treating CKDs.

## Graphical abstract

Tumor protein 53 regulating kinase (TP53RK) is positively correlated to renal dysfunction and fibrosis in chronic kidney disease (CKD). Deleting TP53RK in renal tubule or fibroblasts mitigates renal fibrosis. Mechanically, TP53RK phosphorylates baculoviral IAP repeat containing 5(Birc5) to promote partial epithelial‐mesenchymal transition in renal tubular cells and activation of interstitial fibroblasts. Blockade of TP53RK/Birc5 axis has potential for retarding CKDs.

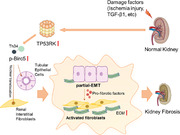

## 1 Introduction

Chronic kidney disease (CKD) is a growing epidemic affecting 9–10% of the population worldwide.^[^ ^1^ ^]^ Renal interstitial fibrosis is a common characteristic of various prevalent CKDs and the extent of interstitial fibrosis is a strong morphologic predictor of clinical outcome.^[^ ^2^ ^]^ It is commonly recognized that myofibroblasts are the protagonists of the fibrosis process and previous research suggests that myofibroblasts may arise from a number of sources such as resident fibroblasts, pericytes, partial epithelial–mesenchymal transition (p‐EMT), endothelial–mesenchymal transition (EndoMT), and bone marrow–derived cells.^[^ ^3^, ^4^, ^5^ ^]^ However, the precise molecular mechanisms underlying the cellular phenotype alteration and activation processes remain poorly understood.

Tumor protein 53 regulating kinase (TP53RK), also known as p53 regulating protein kinase (PRPK), is an atypical protein kinase first found to play a critical role in p53 stabilization by phosphorylation at the *N*‐terminal serine residue Ser15, so as to modulate apoptosis and cell‐cycle arrest.^[^ ^6^ ^]^ TP53RK is also an important subunit of the human cancer testis antigen EKC/KEOPS (endopeptidase‐like kinase chromatin‐associated/kinase, endopeptidase and other proteins of small size) complex, which is a linear structure made of the subunits L Antigen Family Member 3 (LAGE3)‐O‐sialoglycoprotein endopeptidase gene (OSGEP)‐TP53RK‐TP53RK‐Binding Protein (TPRKB).^[^ ^7^ ^]^ The EKC/KEOPS complex functionally affects the accuracy and efficiency of gene transcription and translation by maintaining telomere length^[^ ^8^ ^]^ and regulating the chemical modification of tRNAs.^[^ ^7^ ^]^ In 2017, TP53RK and three other subunit of the KEOPS complex were identified as novel monogenetic causes of Galloway–Mowat syndrome (GAMOS), a renal‐neurological disease characterized by early onset steroid‐resistant nephrotic syndrome in combination with neurodevelopmental defects,^[^ ^9^ ^]^ suggesting a crucial function of TP53RK in the kidney. However, no experimental evidence confirmed the role of TP53RK in CKD.

In this study, our group found a linear correlation between TP53RK expression and kidney fibrosis level and kidney function impairment in CKD patients of various etiologies. Conditional knockout of TP53RK in tubular cells and fibroblasts could both retard renal interstitial fibrosis, indicating that both renal tubular epithelial cells and fibroblasts account for the profibrotic effect of TP53RK. Mechanically, TP53RK might promote p‐EMT of tubular epithelial cells and activate the proliferation of interstitial fibroblasts by phosphorylating baculoviral IAP repeat containing 5 (Birc5) (also known as survivin) at Thr34, which is important for Birc5 stability,^[^ ^10^ ^]^ and facilitates its nuclear translocation; enhanced Birc5 displays a profibrotic effect via activating PI3K/Akt and MAPK pathways. These findings are of great clinical translational potential because pharmacological inhibitors of TP53RK (fusidic acid, FA, an FDA‐approved bacteriostatic antibiotic)^[^ ^11^ ^]^ and Birc5 (YM‐155, an anticancer agent in phase 2 clinical trials)^[^ ^12^ ^]^ have been well developed and both can attenuate renal fibrosis in unilateral ureteral obstruction (UUO) mice. Altogether, our study revealed the critical role of a common TP53RK/Birc5 signaling pathway in promoting both p‐EMT and interstitial fibroblast proliferation during CKD development, and it established the translational potential of TP53RK and Birc5 inhibitors as therapeutic agents against CKDs.

## 2 Results

### 2.1 Upregulated TP53RK was Positively Correlated with Kidney Fibrosis and Kidney Function Impairment

**Figure 1:**
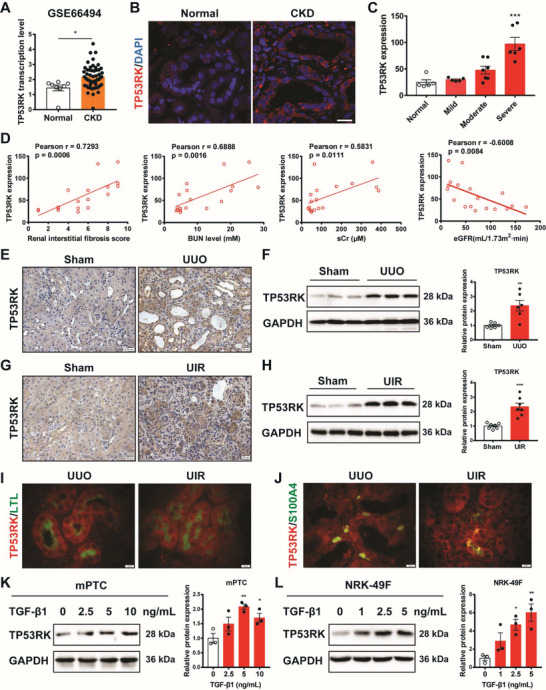
TP53RK was upregulated in kidneys of chronic kidney disease (CKD) patients, kidney fibrosis murine models, and transforming growth factor *β*1 (TGF‐*β*1) induced kidney cells. A) RNA sequencing analysis of human fibrotic kidneys identifies an upregulation of TP53RK expression. Expression of genes was measured using fragments per kilobase million (FPKM). *N* = 8 in normal group and *n* = 53 in CKD group. Data were extracted from https://www.ncbi.nlm.nih.gov/geo, accession: GSE66494. B) Representative immunofluorescence detection of TP53RK expression in biopsy samples from normal individual and CKD patients hospitalized in Department of Nephrology, Children's Hospital of Nanjing Medical University. Scale bar: 10 µm. C) Quantitative analysis of TP53RK expression in hospitalized CKD patients, with mild (*n* = 5), moderate (*n* = 7), or severe (*n* = 6) fibrosis compared to the normal group (*n* = 5) based on immunohistochemical staining. The interstitial fibrosis score was evaluated by a blinded pathologist by estimating the extent of tubular atrophy, infiltration of inflammatory cells, and interstitial fibrosis (0, None; 1, ≤25%; 2, 25–50%; 3, ≥50% for each aspect). Individuals with an interstitial fibrosis score of 1–3 were classified as mild, 4–6 as moderate, and those between 7 and 9 as severe fibrosis. D) Pearson correlation analysis of TP53RK levels and renal interstitial fibrosis scores, blood urea nitrogen (BUN) levels, serum creatinine (sCr) levels and estimated glomerular filtration rates (eGFR) of 18 hospitalized CKD patients. E) Representative immunohistochemical staining of TP53RK in kidneys of unilateral ureteral obstruction (UUO) and sham operated mice (7 days after surgery). Scale bar: 20 µm. F) Immunoblot and semiquantification of TP53RK in kidneys of UUO and sham operated mice at day 7 (*n* = 6). G) Representative immunohistochemical staining of TP53RK in kidneys of unilateral ischemia reperfusion (UIR) and sham operated mice (3 weeks after surgery). Scale bar: 20 µm. H) Representative immunoblotting and semiquantification of TP53RK in day 21 kidneys of UIR and sham operated mice (*n* = 7). I) Immunofluorescence co‐staining of TP53RK and tubular specific marker lotus tetragonolobus lectin (LTL) in UUO and UIR murine kidneys. Red, TP53RK; Green, LTL; Scale bar: 10 µm. J) Immunofluorescence co‐staining of TP53RK and fibroblast specific marker S100A4 in UUO and UIR kidneys. Red, TP53RK; Green, S100A4, Scale bar: 10 µm. K) Protein expression of TP53RK in mouse proximal tubular epithelial cells (mPTCs) treated with TGF‐*β*1 (2.5, 5, and 10 ng mL^−1^) or vehicle (0 ng mL^−1^) for 24 h. L) Protein expression of TP53RK in NRK‐49Fs treated with TGF‐*β*1 (1, 2.5, and 5 ng mL^−1^) or vehicle (0 ng mL^−1^) for 24 h. Data are presented as mean ± SEM; two‐tailed unpaired *t*‐test was used to determine statistical significance of (A), (F) and (H); one‐way ANOVA followed by Dunnett's multiple comparisons test was used to determine statistical significance of (C), (K) and (L); *p < 0.05, ^**^ *p* < 0.01, and ^***^ *p* < 0.001 compared with the normal/sham or vehicle group.

To explore the expression pattern of TP53RK in chronic renal fibrosis, which has never been characterized previously, transcriptional level of TP53RK was analyzed firstly via GEO database (accession: GSE66494). In CKD patients of various etiologies, the average transcription level of TP53RK was significantly upregulated in CKD patients compared to that of the normal group (**Figure** **1**A). Next, the protein expression level of TP53RK was analyzed via immunofluorescence and immunohistochemistry using biopsied kidney tissues from 18 CKD individuals (Table S1, Supporting Information) and five normal individuals hospitalized in our department. Significant upregulation of TP53RK was observed in patient kidneys showing severe fibrosis compared to the normal group (Figure 1B,C, Figure S1A, Supporting Information). The TP53RK protein levels showed positive correlation with renal fibrosis scores, blood urea nitrogen (BUN) levels, and serum creatinine (sCr) levels, and showed negative correlation with estimated glomerular filtration rate (eGFR) (Figure 1D).

The expression of TP53RK was then examined in kidneys of UUO, and unilateral ischemia reperfusion (UIR) murine models, of which UUO is a well‐known model of kidney injury accompanied by progressive tubulointerstitial fibrosis and UIR recapitulates the process of acute kidney injury (AKI)–CKD transition. The sham group manipulated without ligation or clamping served as controls. mRNA expression of TP53RK was more congruously elevated compared to the other three main components of the EKC/KEOPS complex (LAGE3, OSGEP, and TPRKB) in the two distinct murine models (Figure S1B,C, Supporting Information). Time‐lapse transcriptional levels of renal TP53RK in UIR mice were also evaluated at day 1, day 3, and day 21 after surgery by RNA sequencing. Results showed that the level of TP53RK in the UIR group was significantly higher than that of the control group at days 3 and 21 (Figure S1D, Supporting Information), which represents the AKI–CKD phase and CKD phase, respectively.^[^ ^13^ ^]^ Protein levels of TP53RK validated using immunohistochemistry and Western blot showed the same trend (Figure 1E–H). We then verified the expression of TP53RK in proximal tubular cells and interstitial fibroblasts, respectively, using immunofluorescence staining of TP53RK together with proximal tubule marker, lotus tetragonolobus lectin (LTL) or fibroblast‐specific protein S100 calcium binding protein A4 (S100A4).^[^ ^14^, ^15^ ^]^ Results showed that TP53RK was expressed in both tubular epithelial cells and the interstitial fibroblasts of fibrotic kidneys (Figure 1I,J). Moreover, we cultured mouse proximal tubular epithelial cells (mPTCs) and rat kidney fibroblasts (NRK‐49F cells) and stimulated the cells with transforming growth factor *β*1 (TGF‐*β*1) to mimic the pathological conditions of CKD in vitro.^[^ ^16^ ^]^ Accordingly, increasing expression of TP53RK was also observed in both cell lines (Figure 1K,L).

Taken together, TP53RK expression is significantly upregulated both at the transcriptional and protein levels in fibrotic kidneys, and it is associated with the extent of renal fibrosis and kidney function impairment. Also, statistics showed that TP53RK was upregulated in both tubular epithelial cells and interstitial fibroblasts, suggesting a role of TP53RK in activating these two types of cells during CKD progression.

### 2.2 Tubular Deficiency of TP53RK Alleviated Renal Fibrosis

**Figure 2:**
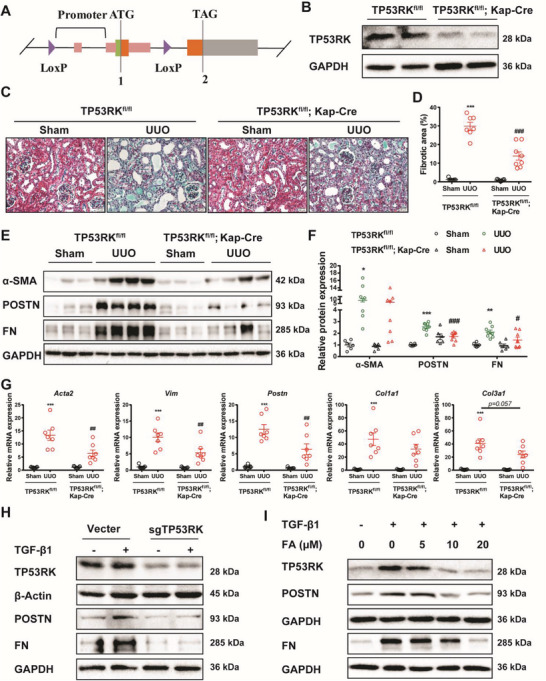
Inhibition of TP53RK alleviated transforming growth factor *β*1 (TGF‐*β*1) induced partial epithelial–mesenchymal transition (p‐EMT) and attenuated unilateral ureteral obstruction (UUO) induced renal fibrosis. A) CRISPR/Cas9 genome engineering was employed to generate floxed TP53RK mice (TP53RK^fl/fl^). B) TP53RK was efficiently deleted in renal tubular of tubular conditional TP53RK knockout mice (TP53RK^fl/fl^; Kap‐Cre). C) TP53RK^fl/fl^ and TP53RK^fl/fl^; Kap‐Cre mice were subjected to sham or UUO operation and euthanized at day 7 in Figure (C–G). Masson's trichrome staining of kidney tissues of corresponding groups. Scale bar: 20 µm. D) Quantification analysis of fibrotic area based on Masson's trichrome staining (*n* = 7–8). E,F) Immunoblot and semiquantification of *α*‐SMA, POSTN, and FN expression (*n* = 6–8). G) Quantitative reverse transcription PCR (qRT‐PCR) analysis of the mRNA level of *Acta2*, *Vim*, *Postn*, *Col1a1*, and *Col3a1* in kidney of corresponding groups (*n* = 7 in each group). H) Control and TP53RK knockdown (KD) mouse proximal tubular epithelial cells (mPTCs) were treated with TGF‐*β*1 (10 ng mL^−1^) for 24 h and harvested for Western blot analysis of TP53RK, POSTN, and FN. Protein semiquantification is presented in Figure S2B (Supporting Information). I) mPTCs were pretreated with vehicle or fusidic acid (FA) (5 × 10^−6^, 10 × 10^−6^, or 20 × 10^−6^ m) 2 h before TGF‐*β*1 (10 ng mL^−1^) treatment for another 24 h. Representative Western blot analyses of TP53RK, POSTN, and FN in each group are shown. Protein semiquantification is presented in Figure S2C (Supporting Information). Data are presented as mean ± SEM; two‐way ANOVA followed by Tukey's multiple comparisons test was used to determine statistical significance of (D), (F), and (G); ^**^ *p* < 0.01, and ^***^ *p* < 0.001 compared with the TP53RK^fl/fl^ + Sham group; ^#^ *p* < 0.05, ^##^ *p* < 0.01, and ^###^ *p* < 0.001 compared with the TP53RK^fl/fl^ + UUO group.

Since both de‐differentiated proximal tubular epithelial cells and activated fibroblasts are candidate cells promoting kidney interstitial fibrosis and TP53RK is highly expressed in these two types of cells, we set out to determine the specific function of TP53RK in each type of cell. For this purpose, we bred mice harboring floxed TP53RK alleles (TP53RK^fl/fl^) (**Figure** **2**A) and generated renal tubular specific and fibroblast specific TP53RK knockout mice by crossing TP53RK^fl/fl^ mice with kidney androgen‐regulated protein (Kap)‐Cre and S100A4‐Cre mice separately. The proximal tubule TP53RK conditional‐knockout (TP53RK^fl/fl^; Kap‐Cre) mice showed almost no expression of TP53RK in extracted tubules (Figure 2B, Figure S2A, Supporting Information). We then analyzed whether kidney injury and scar formation in response to UUO surgery might be affected in TP53RK^fl/fl^; Kap‐Cre mice. Excitingly, Masson's trichrome staining revealed a significantly less extent of tubular brush border loss, tubule atrophy, and tubulointerstitial fibrosis in the kidneys TP53RK^fl/fl^; Kap‐Cre UUO mice compared to that of TP53RK^fl/fl^ UUO mice (Figure 2C,D), in addition to the lower protein expression of alpha smooth muscle actin (*α*‐SMA), periostin (POSTN) and fibronectin (FN) (Figure 2E,F). mRNA level of *Acta2* (which encodes a‐SMA), *Vim* (which encodes vimentin), *Postn*, *Col1a1* (which encodes Collagen I) and *Col3a1* (which encodes Collagen III) showed the same results (Figure 2G). To further illustrate the function of TP53RK in vitro, mPTCs were knocked down (KD) for TP53RK expression using the CRISPR/Cas9 technique (sgTP53RK), followed by TGF‐*β*1 stimulation. We found TP53RK KD dramatically reduced p‐EMT of mPTCs, demonstrated by attenuated expression of POSTN and FN in TP53RK KD cells compared to that of the control cells (Figure 2H; Figure S2B, Supporting Information). Moreover, we tested the effect of FA, a previously reported TP53RK inhibitor,^[^ ^11^ ^]^ in mPTCs. FA was able to downregulate the expression of TP53RK and showed a dose‐dependent protective effect in TGF‐*β*1‐induced mPTCs (Figure 2I, Figure S2C, Supporting Information). FA treatment had similar effect as TP53RK KD in mPTCs under the experimental conditions with single or double interventions, indicating that the effects of FA were largely dependent on TP53RK inhibition (Figure S2D,E, Supporting Information). These results indicate that tubular TP53RK suppression may function to alleviate interstitial fibrosis by antagonizing p‐EMT of tubular epithelial cells.

### 2.3 Fibroblast Deficiency of TP53RK Showed a Fibrotic Alleviating Effect

**Figure 3:**
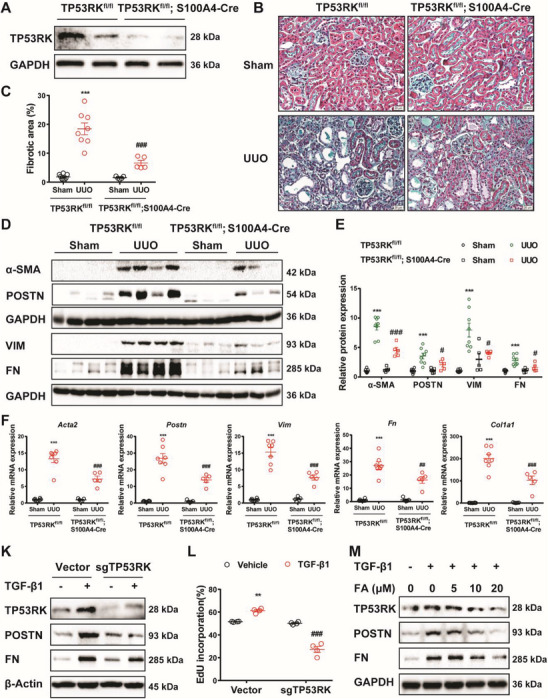
Genetic and pharmaceutic inhibition of TP53RK alleviated transforming growth factor *β*1 (TGF‐*β*1) induced renal interstitial fibroblasts activation and attenuated unilateral ureteral obstruction (UUO) induced renal fibrosis. A) TP53RK was efficiently deleted in renal interstitial fibroblasts of conditional TP53RK knockout mice (TP53RK^fl/fl^; S100A4‐Cre). B) TP53RK^fl/fl^ and TP53RK^fl/fl^; S100A4‐Cre mice of were subjected to sham or UUO operation and euthanized 7 days after establishment of the model in Figure B–F. Masson's trichrome staining of kidney tissues of mice in corresponding groups. Scale bar: 20 µm. C) Quantification analysis of fibrotic area based on Masson's trichrome staining (*n* = 5–8). D,E) Western blot and semiquantification of *α*‐SMA, POSTN, VIM, and FN expression (*n* = 5–8). F) mRNA levels of *Acta2*, *Postn*, *Vim*, *Fn*, and *Col1a1* in kidney of corresponding groups (*n* = 5–7). K) Control (vector) and TP53RK knockdown (sgTP53RK) NRK‐49Fs were treated with vehicle or TGF‐*β*1 (5 ng mL^−1^) for 24 h. Immunoblots of TP53RK, POSTN, and FN are shown. Protein semiquantification is presented in Figure S3B (Supporting Information) (*n* = 3). L) Control (vector) and TP53RK KD NRK‐49F cells (sgTP53RK) were treated as in (K). Cells were stained for EdU and Hoechst 33342. The EdU incorporation rate was quantified by normalizing the number of EdU positive cells against total cell nucleus number (*n* = 4). Representative images are shown in Figure S3D (Supporting Information). M) NRK‐49Fs were pretreated with vehicle or FA (5 × 10^−6^, 10 × 10^−6^, or 20 × 10^−6^ m) 2 h before TGF‐*β*1 (5 ng mL^−1^) treatment for another 24 h. Representative Western blot analyses of TP53RK, POSTN, and FN in each group are shown. Protein semiquantification is presented in Figure S3E (Supporting Information) (*n* = 3). Data are presented as mean ± SEM; two‐way ANOVA followed by Tukey's multiple comparisons test was used to determine statistical significance of (C), (E), (F), and (L); ^***^ *p* < 0.001 compared with the TP53RK^fl/fl^ + Sham or vector + vehicle group; ^#^ *p* < 0.05, ^##^ *p* < 0.01, and ^###^ *p* < 0.001 compared with the TP53RK^fl/fl^ + UUO or vector + TGF‐*β*1 group.

We generated fibroblast conditional TP53RK knockout (TP53RK^fl/fl^; S100A4‐Cre) mice with approximately 90% reduction in cultured primary renal interstitial fibroblasts, compared with those of TP53RK^fl/fl^ mice (**Figure** **3**A, Figure S3A, Supporting Information). With the UUO model, we found that fibroblast conditional TP53RK knockout significantly attenuated the tubulointerstitial fibrotic pathology extent in the kidneys (Figure 3B,C). Consistently, the protein (*α*‐SMA, POSTN, VIM, and FN) (Figure 3D,E) and mRNA (*Acta2*, *Postn*, *Vim*, *Fn*, and *Col1a1*) (Figure 3F) expression levels of fibrotic factors were also significantly lower in TP53RK^fl/fl^; S100A4‐Cre UUO mice compared to TP53RK^fl/fl^ UUO mice. Furthermore, TP53RK was also KD in rat kidney fibroblasts (NRK‐49Fs) cell line and stimulated with TGF‐*β*1. Consistent to the fibrosis alleviating effect in vivo, a dramatically reduced protein (POSTN and FN) (Figure 3K, Figure S3B, Supporting Information) and mRNA (*Acta2*, *Vim*, *Fn*, *Col1a1* and *Col3a1*) (Figure S3C, Supporting Information) expression of fibroblast activation markers was seen in response to TP53RK KD. Cell proliferation of NRK‐49Fs under TGF‐*β*1 stimulation was also alleviated upon TP53RK KD as demonstrated by a decreased EdU incorporation rate (Figure S3D, Supporting Information , Figure 3L). Also, FA dose‐dependently downregulated TP53RK expression and alleviated TGF‐*β*1‐induced NRK‐49Fs activation (Figure 3M, Figure S3E,F, Supporting Information). In NRK‐49Fs, FA treatment again showed similar protective effect as TP53RK KD upon TGF‐*β*1 treatment. Moreover, FA treatment did not show a further decrease in FN expression in TP53RK KD NRK‐49Fs, indicating that the protective effect of FA was dependent on TP53RK inhibition (Figure S3G,H, Supporting Information). Thus, suppressing TP53RK also functioned to alleviate kidney interstitial fibrosis by inhibiting the activation and proliferation of fibroblasts.

### 2.4 Global TP53RK Overexpression Aggravated, While Global TP53RK Knockdown Alleviated UUO‐Induced Kidney Fibrosis

Because we found a consistent effect of TP53RK in tubules as well as in fibroblasts, we then investigated the function of TP53RK by global delivery of TP53RK overexpression and knockdown plasmids utilizing hydrodynamic‐based tail vein plasmid delivery approach, which is previously proved to be viable for manipulating gene expression globally.^[^ ^17^, ^18^, ^19^ ^]^ Experiment data showed that, the concentrations of aspartate aminotransferase (AST), alanine aminotransferase (ALT), BUN, sCr, lactate dehydrogenase (LDH), and creatine kinase myocardial band (CK‐MB) in the serum of mice receiving hydrodynamic‐based tail vein delivery of plasmids showed no obvious difference from those of mice receipting tail vain delivery of 200 µL saline 1 week after injection, indicating lack of damage to the liver, kidney, and heart (Figure S4A–F, Supporting Information). Additionally, tissue histology further supported no significant injury in liver, kidney, heart, and lung tissues (Figure S4G, Supporting Information). Using hydrodynamic‐based tail vein plasmid delivery method, we firstly strengthened the global expression of TP53RK in mice. Thirty‐six hours after the plasmid delivery, mice were subjected to UUO surgery and sacrificed 7 days thereafter. We found globally TP53RK overexpression profoundly aggravated tubule brush border loss, tubule atrophy, and tubulointerstitial matrix deposition in the UUO kidneys (Figure S5A,B, Supporting Information). Whole kidney protein level analysis showed that hydrodynamic‐based tail vein injection for only once was able to increase the kidney expression of TP53RK until the end of the experiment and that overexpression of TP53RK was able to decrease the protein expression of *α*‐SMA, VIM, and FN (Figure S5C,D, Supporting Information). Taken together, these data indicated that global overexpression of TP53RK resulted in significantly aggravated kidney fibrosis.

We then knocked down TP53RK by hydrodynamic‐based tail vein delivery of CRISPR/Cas9 plasmid to investigate whether it could in turn alleviate UUO‐induced kidney fibrosis in mice. After injecting with the CRISPR/Cas9 plasmid for 72 h, protein expression of TP53RK in kidney was effectively knocked down to 55.8% (Figure S6A, Supporting Information). Based on this, we carried out UUO surgery 36 h after hydrodynamic‐based tail vein injection. One week after establishing the UUO model, mice were sacrificed. As expected, TP53RK knockdown in mice remarkably alleviated tubule injury and tubulointerstitial fibrosis in UUO kidneys (Figure S6B,C, Supporting Information). Accordingly, the protein expression levels of *α*‐SMA, VIM, and FN, which was obvious in UUO mice injected with the vector, was downregulated in response to TP53RK KD (Figure S6D,E, Supporting Information). Together, by utilizing tubular conditional TP53RK knockout and fibroblast conditional TP53RK knockout mice, global TP53RK overexpression and knockdown mice, as well as in vitro cell models, we found a profibrotic effect of TP53RK in CKD interstitial fibrosis.

### 2.5 TP53RK Phosphorylated and Mediated Nuclear Localization of Birc5

**Figure 4:**
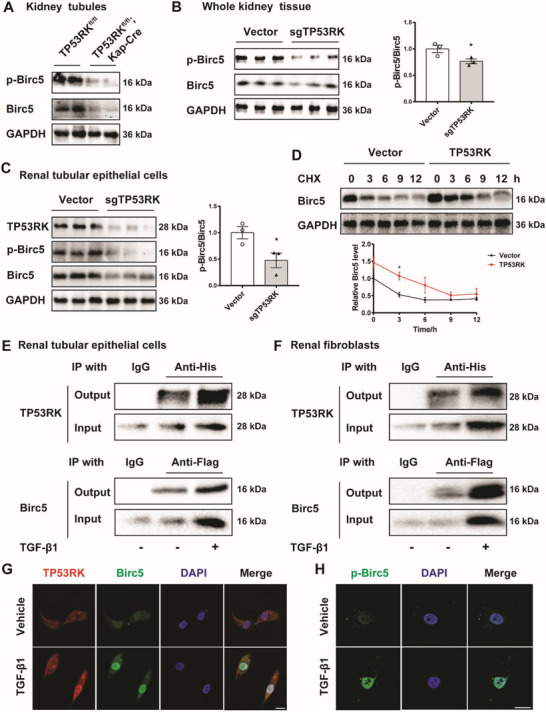
TP53RK phosphorylated and increased the nuclear localization of Birc5. A) Renal tubular of tubular conditional TP53RK knockout mice (TP53RK^fl/fl^; Kap‐Cre) was separated and p‐Birc5 (Thr34) and Birc5 expression were estimated with Western blot. B) Mice were subjected to hydrodynamic‐based tail vein injection of TP53RK targeted CRISPR/Cas9 plasmid. p‐Birc5 and Birc5 expression in the kidneys were estimated 72 h after injection (*n* = 3). The ratio of p‐Birc5 against Birc5 was calculated. C) TP53RK expression was knocked down in mouse proximal tubular epithelial cells (mPTCs) with CRISPR/Cas9 technique. The expression of TP53RK, p‐Birc5, and Birc5 was measured with Western blot (*n* = 3). The ratio of p‐Birc5 against Birc5 was calculated. D)mPTCs were transfected with vector or TP53RK overexpression plasmids. Twenty‐four hours after transfection, cells were treated with CHX (10 ng mL^−1^) and collected at 0, 3, 6, 9, and 12 h. The protein level of Birc5 was measured with Western blot (*n* = 3). E) mPTCs were co‐transfected with TP53RK‐Flag and Birc5‐His plasmids and treated with vehicle or transforming growth factor *β*1 (TGF‐*β*1) (10 ng mL^−1^) for 24 h before Co‐IP analysis of interaction between the TP53RK‐Flag and Birc5‐His fusion proteins. Anti‐His and anti‐Flag antibodies were used for immunoprecipitating. F) NRK‐49Fs were co‐transfected with TP53RK‐Flag and Birc5‐His and treated with vehicle or TGF‐*β*1 (5 ng mL^−1^) for 24 h before Co‐IP analysis of interaction between the TP53RK‐Flag and Birc5‐His fusion proteins. Anti‐His and anti‐Flag antibodies were used for immunoprecipitating. G) Immunofluorescence analysis of TP53RK (red fluorescence) and Birc5 (green fluorescence) in human proximal tubular epithelial cells (HK2) cells treated with vehicle or TGF‐*β*1 (10 ng mL^−1^). The nucleus was stained with 4, 6‐diamidino‐2‐phenylindole (DAPI, blue fluorescence). Scale bar, 20 µm. H) Immunofluorescence analysis of p‐Birc5 (green fluorescence) in HK2 cells treated with vehicle or TGF‐*β*1 (10 ng mL^−1^); the nucleus was stained with DAPI (blue fluorescence); scale bar, 20 µm. Data are presented as mean ± SEM; two‐tailed unpaired *t*‐test was used to determine statistical significance of (B), (C) and (D); *p < 0.05 compared with the Vector group.

Although TP53RK was initially found to regulate the tumor suppressor p53 and mediated its phosphorylation on Ser15,^[^ ^6^ ^]^ a more recently published study revealed that TP53RK did not directly bind to p53. Instead, TP53RK bound to and phosphorylated Birc5 at Thr34 and mediated colon cancer metastasis.^[^ ^11^ ^]^ In line with this reference, we overexpressed and knocked down TP53RK in mPTCs and found no influence on p53 phosphorylation on Ser15 (Figure S7, Supporting Information). Instead, the expression and phosphorylation of Birc5 (Thr34) were greatly downregulated in kidney tubule of TP53RK^fl/fl^; Kap‐Cre mice, kidney of global TP53RK KD mice and TP53RK KD mPTCs (**Figure** **4**A–C), with the ratio of p‐Birc5/Birc5 significantly downregulated (Figure 4B–C). Since it is reported that the phosphorylation of Birc5 at Thr34 is important for Birc5 stability,^[^ ^10^ ^]^ we then evaluated the stability of Birc5 protein after TP53RK overexpression. Protein synthesis inhibitor cycloheximide (CHX) was used for monitoring protein stability. As shown in Figure 4D, TP53RK overexpression significantly strengthened the expression level of Birc5 in mPTCs. After treating with CHX, endogenous Birc5 was degraded with a half‐life of ≈3 h in vector group while TP53RK overexpression significantly extended the half‐life of Birc5 to about 7 h. These results added to the evidence that TP53RK phosphorylated Birc5 at Thr34 and increased its protein stability. Notably, co‐immunoprecipitation (Co‐IP) analysis in both mPTCs and NRK‐49Fs overexpressing TP53RK‐Flag and Birc5‐His fusion proteins revealed that TP53RK‐Flag directly associated with Birc5‐His protein, and that binding was strengthened by TGF‐*β*1 stimuli (Figure 4E,F). Immunofluorescent staining showed that under normal conditions, both TP53RK and Birc5 molecules colocalized in the cytoplasm, while in response to TGF‐*β*1 treatment, both molecules increased and Birc5 tended to localize within nucleus (Figure 4G). This might be because Birc5 is increasingly phosphorylated at Thr34 by TP53RK in response to TGF‐*β*1 treatment, and p‐Birc5 is primarily localized in the nucleus (Figure 4H). These results demonstrate that the profibrotic effect of the kinase TP53RK may be mediated by phosphorylating downstream Birc5.

**Figure 5:**
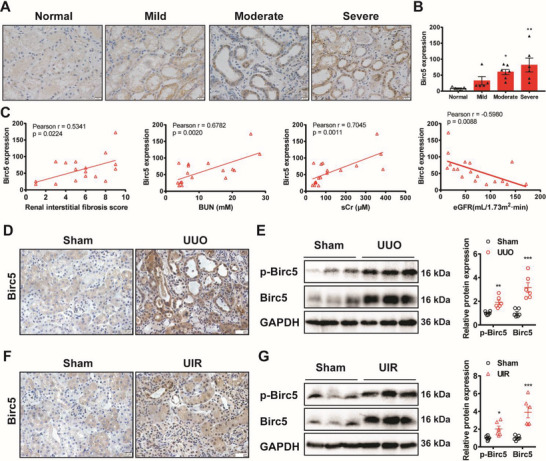
Birc5 was upregulated in fibrotic kidneys. A) Representative immunohistochemical staining of Birc5 expression in biopsy samples from chronic kidney disease (CKD) patients hospitalized in Department of Nephrology, Children's Hospital of Nanjing Medical University (scale bar, 20 µm). The interstitial fibrosis score was evaluated by a blinded pathologist by estimating the extent of tubular atrophy, infiltration of inflammatory cells, and interstitial fibrosis (0, None; 1, ≤25%; 2, 25–50%; 3, ≥50% for each aspect). Individuals with an interstitial fibrosis score of 1–3 were classified as mild, 4–6 as moderate, and those between 7 and 9 as severe fibrosis. B) Quantitative analysis of Birc5 expression in CKD patients with mild (*n* = 5), moderate (*n* = 7), or severe (*n* = 6) fibrosis compared to the normal group (*n* = 5) based on immunohistochemical staining. C) Pearson correlation analysis of Birc5 levels and renal interstitial fibrosis scores, blood urea nitrogen (BUN) levels, serum creatinine (sCr) levels and eGFR levels in CKD patients (*n* = 18 in total). D) Immunohistochemical staining of Birc5 in kidneys 7 days after unilateral ureteral obstruction (UUO) and sham operation (scale bar, 20 µm). E) Immunoblot and semiquantification of Birc5 and p‐Birc5 in kidneys of UUO and sham operated mice at day 7 (*n* = 6). F) Immunohistochemical staining of Birc5 in kidneys 21 days after unilateral ischemia reperfusion (UIR) and sham operation (scale bar, 20 µm). G) Western blot analysis and semiquantification of Birc5 and p‐Birc5 in day 21 kidneys of UIR and sham operated mice (*n* = 6). Data are presented as mean ± SEM; one‐way ANOVA followed by Dunnett's multiple comparisons test was used to determine statistical significance of (B); two‐tailed unpaired *t*‐test was used to determine statistical significance of (E) and (G); *p < 0.05, ^**^ *p* < 0.01, and ^***^ *p* < 0.001 compared with the normal or sham group.

Because the role of Birc5 in CKD has not been fully illustrated in CKD before, we first analyzed the expression of Birc5 in biopsy of CKD patients, as well as in CKD murine models. Compared to the normal group, protein levels of Birc5 were also significant upregulated with the progression of kidney fibrosis in CKD patients (**Figure** **5**A,B). Semiquantification based on the immunohistochemistry staining showed that Birc5 protein levels were positively correlated with kidney fibrosis scores, BUN levels, and sCr levels and negatively correlated with eGFR of CKD patients (Figure 5C). Accumulation of Birc5 and its phosphorylated form (at Thr34) was also observed in the kidneys of UUO and UIR mice via immunohistochemistry staining and Western blotting (Figure 5D–G). Notably, a clear nucleus location of Birc5 was seen both in CKD patients and murine models (Figure 5A,D,F). Thus, these data strongly suggest that Birc5 might function in the downstream signaling of TP53RK in promoting kidney fibrosis.

### 2.6 Birc5 Knockdown Attenuated TGF‐*β*1‐Induced Profibrotic Phenotypes in Renal Tubular Epithelial Cells, Renal Interstitial Fibroblasts, and UUO‐Induced Kidney Fibrosis

**Figure 6:**
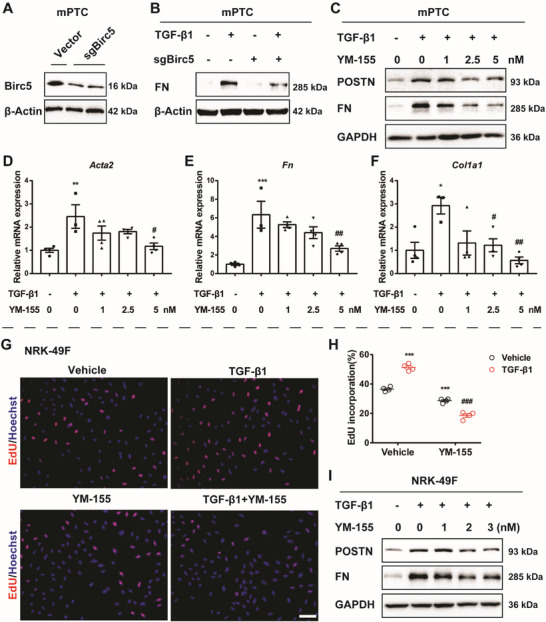
Genetic and pharmaceutical inhibition of Birc5 retarded profibrotic phenotypes in renal tubular epithelial cells and renal interstitial fibroblasts. A) Mouse proximal tubular epithelial cells (mPTCs) were transfected with vector or Birc5 targeting CRISPR/Cas9 plasmid (sgBirc5). Birc5 KD efficiency was evaluated by Western blot 24 h after transfection. B) Control (vector) and Birc5 KD (sgBirc5) mPTCs were treated with vehicle or transforming growth factor *β*1 (TGF‐*β*1) (10 ng mL^−1^) for 24 h and harvested for Western blot analysis of FN. (C) mPTCs were pretreated with YM‐155 (1 × 10^−9^, 2.5 × 10^−9^, or 5 × 10^−9^ m) for 2 h and stimulated with vehicle or TGF‐*β*1 (10 ng mL^−1^) for 24 h and harvested for Western blotting analysis of POSTN and FN. (D–F) mPTCs were treated as is described in (C) and harvested for qRT‐PCR analysis of *Acta2*, *Fn*, and *Col1a1* (*n* = 3). (G–H) NRK‐49Fs were pretreated with vehicle or YM‐155 (1 × 10^−9^, 2 × 10^−9^, or 3 × 10^−9^ m) 2 h before TGF‐*β*1 (5 ng mL^−1^) treatment for another 24 h. Cells were stained for EdU and Hoechst 33342. The EdU incorporation rate was quantified by normalizing the number of EdU positive cells against total cell nucleus number (*n* = 4). (I) Cells were treated as in (G–H) and harvested for Western blotting. Representative blots of POSTN and FN are shown. Data are presented as mean ± SEM; one‐way ANOVA followed by Dunnett's multiple comparisons test was used to determine statistical significance of (D–F); two‐way ANOVA followed by Tukey's multiple comparisons test was used to determine statistical significance of (H); *p < 0.05, ^**^ *p* < 0.01 and ^***^ *p* < 0.001 compared with the vehicle group; ^#^ *p* < 0.05, ^##^ *p* < 0.01 and ^###^ *p* < 0.001 compared with the vehicle+TGF‐*β*1 group.

To test the hypothesis mentioned above, we further tested the interference of Birc5 expression in renal tubular epithelial cells and renal interstitial fibroblasts. In mPTCs, both genetically knocking down Birc5 and pharmaceutical inhibition of Birc5 by its selective inhibitor YM‐155 ^[^ ^12^ ^]^ could markedly suppress the TGF‐*β*1‐induced cellular p‐EMT (**Figure** **6**A–F). In NRK‐49Fs, YM‐155 treatment significantly blunted cell proliferation induced by TGF‐*β*1 (Figure 6G,H). Birc5 inhibition also resulted in decreased protein expression levels of POSTN and FN (Figure 6I), demonstrating that suppressing Birc5 exerted an antiactivation effect on renal interstitial fibroblasts in the context of TGF‐*β*1 stimuli.

**Figure 7:**
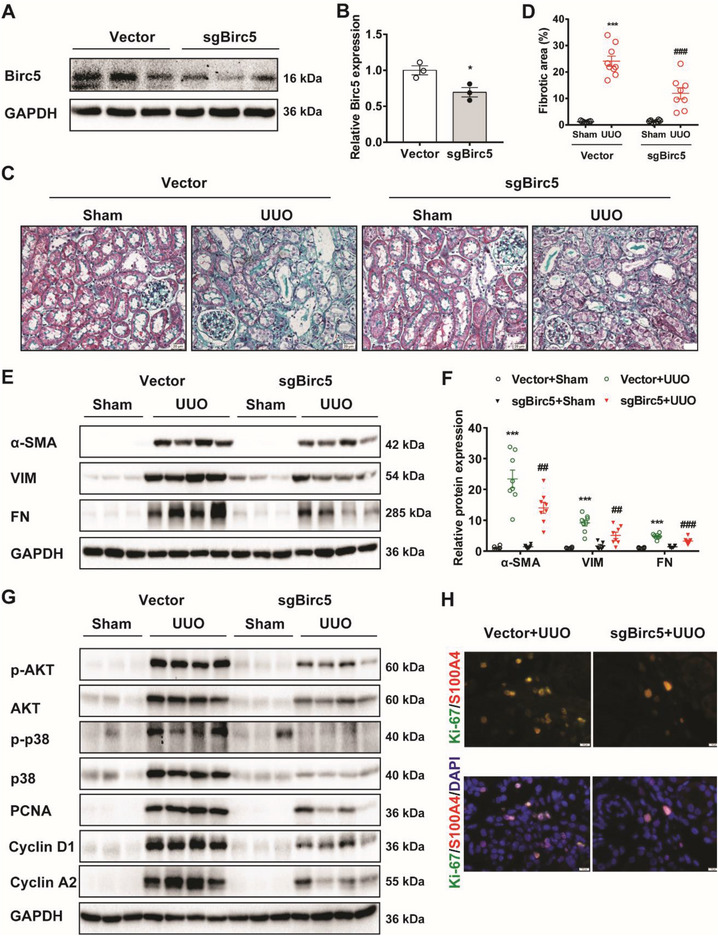
Birc5 knockdown mitigated unilateral ureteral obstruction (UUO) induced renal fibrosis and (myo)fibroblasts proliferation. (A‐B) Mice were knockdown for Birc5 expression via hydrodynamic‐based tail vein delivery of the Birc5 targeted CRISPR/Cas9 plasmid (sgBirc5). Western blot (A) and semiquantification of Birc5 expression in kidneys of each group were shown (B) (*n* = 3). (C–D) In Figure (C–H), mice injected with empty vector or sgBirc5 plasmid were subjected to UUO or sham operation and euthanized 7 days after establishment of the model. Representative images of Masson's trichrome staining (C) and quantification of fibrotic area (D) were shown (*n* = 7–9). Scale bar, 20 µm. E,F) Western blot analysis and semiquantification of *α*‐SMA, VIM, and FN expression in kidney tissues (*n* = 6–8). G) Representative immunoblots of p‐AKT, AKT, p‐p38, and p38, PCNA, cyclin D1, and cyclin A2 expression in kidney tissues. Protein semiquantification is presented in Figure S8E,F (Supporting Information). H) Immunofluorescent staining of Ki‐67 and S100A4 in UUO mice injected with vector and sgBirc5 plasmid. Red, S100A4; Green, Ki‐67; blue, DAPI; scale bar, 10 µm. Data are presented as mean ± SEM; two‐tailed unpaired *t*‐test was used to determine statistical significance of (B); two‐way ANOVA followed by Tukey's multiple comparisons test was used to determine statistical significance of (D) and (F); *p < 0.05 and ^***^ *p* < 0.001 compared with the vector or vector + Sham group; ^##^ *p* < 0.01 and ^###^ *p* < 0.01 compared with the vector + UUO group.

We next evaluated the role of Birc5 in UUO mice by hydrodynamic‐based tail vein delivery of the Birc5 targeting CRISPR/Cas9 plasmid(sgBirc5). Expression of Birc5 in kidney tissues was measured 72 h after plasmid delivery. As shown in **Figure** **7**A,B, Birc5 expression levels were markedly decreased with this method. Consistent with the effect of TP53RK in UUO mice, Birc5 KD markedly improved renal pathological damage in UUO mice compared to that of mice injected with empty vector, with less severe tubular atrophy and tubulointerstitial fibrosis (Figure 7C,D). Also, the upregulated protein expression patterns of *α*‐SMA, VIM, and FN in the UUO kidneys were all efficiently reversed by Birc5 KD (Figure 7E,F).

### 2.7 TP53RK/Birc5 may Promote a Fibrosis Response by Promoting Aberrant Proliferation of (myo)Fibroblasts

Given the vital role of myofibroblast proliferation in kidney fibrosis, we speculated that proliferation‐related pathways likely participate in the TP53RK/Birc5‐mediated pro‐fibrotic effect. PI3K/Akt and MAPK signaling pathways have been reported to play key roles in promoting myofibroblast proliferation.^[^ ^20^ ^]^ Consistently, our results also revealed remarkably increased activity of MAPK and PI3K/Akt signaling pathways in the UUO kidneys (Figure 7G). Moreover, our in vitro experiments showed that Birc5 overexpression significantly upregulated, while Birc5 knocking down remarkably downregulated both total and phosphorylated AKT and p38 in mPTCs (Figure S8A,B, Supporting Information). Same results were gained in NRK‐49Fs (Figure S8C,D, Supporting Information). In UUO mice, Birc5 knockdown with the CRISPR/Cas9 plasmid efficiently inhibited excessive activation of PI3K/Akt and MAPK pathways (Figure 7G, Figure S8E, Supporting Information). These data suggest that PI3K/Akt and MAPK pathways are the downstream signaling of Birc5. In addition, the aberrant upregulation of proliferating cell nuclear antigen (PCNA) and cell cycle regulator cyclin D1 and cyclin A2 in UUO was also attenuated in Birc5 KD mice (Figure 7G, Figure S8F, Supporting Information). Consistently, immunofluorescent staining of Ki‐67 (a proliferation marker) and (myo)fibroblasts marker S100A4 indicated that Birc5 KD efficiently decreased the ratio of Ki‐67 and S100A4 positive cells in tubulointerstitial region of UUO kidneys (Figure 7H). Taken together, TP53RK/Birc5 may promote a fibrosis response by activating the downstream PI3K/Akt and MAPK signaling pathways in myofibroblasts.

### 2.8 Tubular Conditional Knockout of TP53RK Attenuated AKI–CKD Transition

Because AKI is a key risk factor for the development of CKD,^[^ ^13^ ^]^ we next examined whether TP53RK knockout in the tubule could also attenuate AKI–CKD transition. A 45‐min UIR mouse model was adopted and kidney tissues were collected 3 weeks after UIR. When compared with the TP53RK^fl/fl^ + UIR group, the tubulointerstitial damage, including tubular atrophy, flattening, and sloughing of tubular epithelial cells, cast formation, and extracellular matrix accumulation, were dramatically ameliorated by tubular conditional TP53RK knockout (Figure S9A,B, Supporting Information). Notably, tubular conditional knockout of TP53RK also reduced the protein levels of *α*‐SMA, VIM, and FN in the kidneys at day 21 after UIR injury (Figure S9C,D, Supporting Information). Consistently, the upregulated mRNA levels of fibrotic markers (*Acta2*, *Vim*, *Postn*, *Fn*, *Col1a1*, and *Col3a1*) induced by UIR were also attenuated in TP53RK^fl/fl^; Kap‐Cre mice compared to those of TP53RK^fl/fl^ + UIR mice (Figure S9E, Supporting Information). These data indicate that genetic ablation of TP53RK in the tubule can also interrupt kidney fibrosis after AKI.

### 2.9 TP53RK/Birc5 as Therapeutic Targets to Retard Renal Fibrosis in CKD

**Figure 8:**
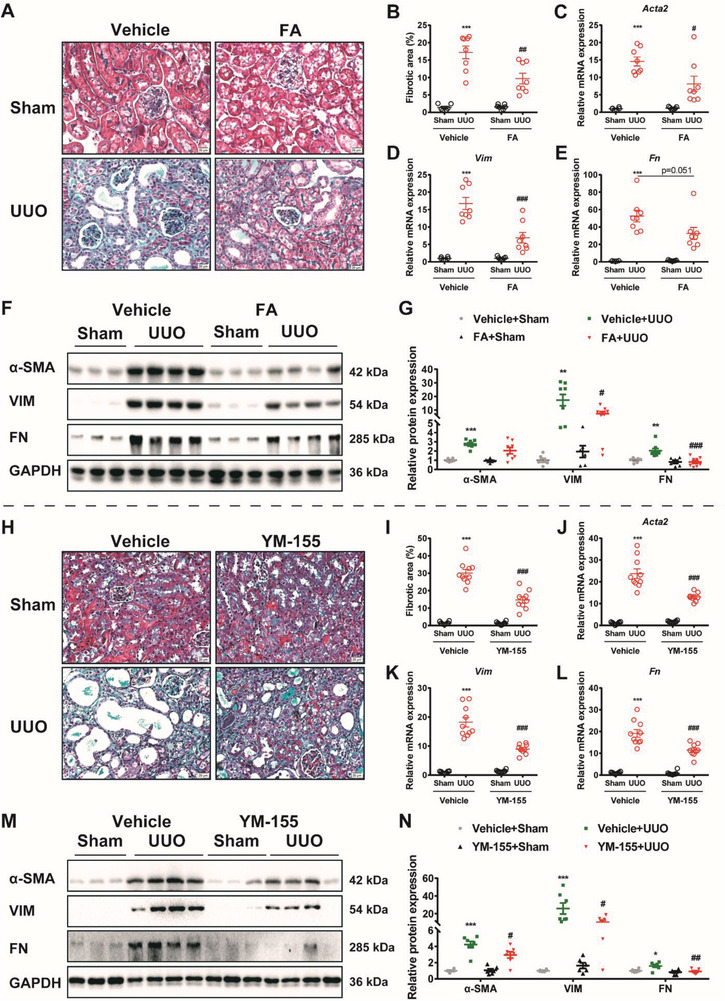
TP53RK/Birc5 as a potential therapeutic targets in renal fibrosis. A,B) In Figures (A–G), mice were pretreated with TP53RK inhibitor fusidic acid (FA, 16 mg kg^−1^ d^−1^) and subjected to sham or unilateral ureteral obstruction (UUO) operation and euthanized 7 days after establishment of the model. Deposition of extracellular matrix in kidney tissues was determined by Masson's trichrome staining (A) (scale bar, 20 µm). Quantification analysis of fibrotic area was shown in parallel (B) (*n* = 6–8). C–E) Kidney tissues were collected and subjected to qRT‐PCR analysis of *Acta2*, *Vim*, and *Fn* (*n* = 6–8). F) Representative Western blot and semiquantification of *α*‐SMA, VIM, and FN expression in kidney tissues of corresponding group (*n* = 6–8). H–I) In Figures H–N, mice were pretreated with Birc5 selective inhibitor YM‐155 (3 mg kg^−1^ d^−1^) and subjected to sham or UUO operation and euthanized 14 days thereafter. Representative Masson's trichrome staining (H) and quantification of fibrotic area (I) were shown (*n* = 9–10). Scale bar: 20 µm. J–L) mRNA level of *Acta2*, *Vim*, and *Fn* in corresponding group (*n* = 9–10). M) Representative immunoblots of *α*‐SMA, VIM, and FN expression. N) Semiquantification of *α*‐SMA, VIM, and FN expression in YM‐155 treated UUO mice (*n* = 6–7). Data are presented as mean ± SEM; two‐way ANOVA followed by Tukey's multiple comparisons test was used to determine the statistical significance; *p < 0.05, ^**^ *p* < 0.01 and ^***^ *p* < 0.001 compared with the vehicle + Sham group; ^#^ *p* < 0.05, ^##^ *p* < 0.01 and ^###^ *p* < 0.001 compared with the vehicle + UUO group.

The data above led us to investigate the therapeutic potential of retarding renal fibrosis by targeting TP53RK/Birc5 axis. Fortunately, FA is an FDA‐approved antibiotic ^[^ ^11^ ^]^ and YM‐155 is under phase 2 clinical trials in patients of lymphoma, lung cancer, breast cancer, prostate cancer, non‐small cell lung cancer (NSCLC), and melanoma.^[^ ^21^, ^22^, ^23^, ^24^, ^25^ ^]^ The efficacy of FA was first verified in UUO mice. Mice were pretreated with FA (16 mg kg^−1^) before UUO surgery and then treated daily for 7 consecutive days and sacrificed. Masson's trichrome staining of kidneys from FA‐treated mice revealed a recovered morphology with a low degree of fibrotic deposition on day 7 after UUO (**Figure** **8**A,B). Consistently, the mRNA and protein levels of *α*‐SMA, VIM, and FN (Figure 8C–G) were also significantly lower in kidneys of FA‐treated mice. In parallel, the antifibrotic effect of YM‐155 was also tested in vivo. YM‐155 (3 mg kg^−1^) was given to mice via i.p. 24 h before UUO surgery and then treated daily for 14 consecutive days before mice were sacrificed. Data analysis from Masson's trichrome staining, quantitative reverse transcription PCR (qRT‐PCR), and Western blotting all point to the improved renal conditions of YM‐155‐treated UUO mice compared to that of vehicle‐treated UUO mice (Figure 8H–N). However, we could not see synergistic renal protective effects when combining the two inhibitors together both in *vivo* and in *vitro* (Figure S10, Supporting Information).

## 3 Discussion

**Figure 9:**
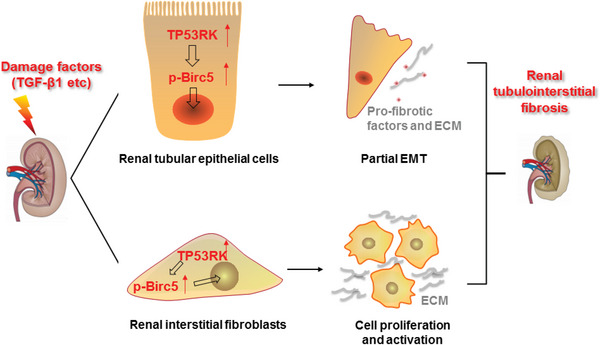
Schematic diagram illustrating the profibrotic role of TP53RK in kidney. In response to damage factors such as transforming growth factor *β*1 (TGF‐*β*1), expression of TP53RK in renal tubular epithelial cells and interstitial fibroblasts are both upregulated. Strengthened expression of TP53RK phosphorylates and stabilizes downstream Birc5 and facilitates its nuclear translocation. Enhanced Birc5 mediates partial epithelial–mesenchymal transition (p‐EMT) of renal tubular epithelial cells, proliferation, and activation of renal interstitial fibroblasts, and finally drives chronic kidney disease (CKD) progression.

In this study, we demonstrated that TP53RK, an atypical protein kinase and an important component of the EKC/KEOPS complex, contributed critically to the pathogenesis of renal fibrosis. TP53RK was upregulated in kidneys of CKD patients and renal fibrosis murine models induced by UUO and UIR. Global overexpression of TP53RK aggravated UUO‐induced kidney fibrosis, while TP53RK knockdown with the CRISPR/Cas9 plasmid alleviated UUO‐induced kidney fibrosis. Specific genetic deletion of *TP53RK* either in renal tubules or in fibroblasts could mitigate renal fibrosis in mice models. In vitro, TP53RK KD or inhibition by FA reversed TGF‐*β*1‐mediated p‐EMT of renal tubular epithelial cells and proliferation and activation of renal interstitial fibroblasts. We found that overexpressing or ablating TP53RK posed no effect on the expression of fibrotic markers such as *α*‐SMA, VIM, and FN both in vitro and in vivo under basal conditions. However, under CKD conditions, modulating TP53RK expression significantly influenced the extent of fibrosis, with *α*‐SMA, VIM, and FN expression as markers for evaluating fibrosis extent. These results suggested that these up‐mentioned fibrotic markers are not the direct downstream targets of TP53RK. Instead, investigations showed that TP53RK may promote renal fibrogenesis by promoting the phosphorylation and nuclear translocation of Birc5, thus enhancing proliferation of (myo)fibroblasts possibly via activating PI3K/Akt and MAPK signaling pathways. The upregulated expression of Birc5 and the profibrotic effect of Birc5 exactly paralleled that of TP53RK. Taken together, these results reveal a novel causal role of upregulated TP53RK, which correlated well with renal fibrosis and kidney function impairment, in promoting renal fibrosis through phosphorylation and nuclear translocation of Birc5 and thus enhancing cellular phenotypic alterations in both renal tubular epithelial cells and fibroblasts. Especially noteworthy is that targeting the TP53RK/Birc5 axis to retard renal fibrosis is of high translational probability, because the TP53RK inhibitor FA is a clinically commonly used antibiotic and Birc5 selective inhibitor YM‐155 is currently in clinical phase 2 trials. A schematic in **Figure** **9** illustrates the profibrogenic function of TP53RK in kidney.

Numerous efforts have aimed to discover the precursors of the matrix‐producing myofibroblast cells in the fibrosis process of kidney and key candidate cells that have been proposed include tubular epithelial cells, fibroblasts, pericytes, hematopoietic cells, and bone marrow–derived cells.^[^ ^3^, ^4^, ^5^ ^]^ Tubular epithelial cells account for the largest number of cells in the cortex of the kidney and are particularly vulnerable to injury in a variety of CKDs. Although it is debated whether p‐EMT accounts for a minor contribution to the scar‐producing cells, a number of research reported that targeted regulation on key signaling niches,^[^ ^26^, ^27^, ^28^ ^]^ cell metabolism,^[^ ^29^, ^30^ ^]^ and secretion of exosome^[^ ^31^, ^32^ ^]^ and profibrotic factors^[^ ^33^, ^34^ ^]^ of kidney epithelial cells, indeed influenced the extent of kidney fibrosis. Thus, we might conclude that although evidence do not support the direct transformation of kidney epithelial cells to myofibroblasts, injured and transformed epithelial cells play an important role in promoting the proliferation and activation myofibroblasts in autocrine and paracrine manners. Our findings provided convincing evidence that by hampering the profibrotic phenotype of epithelial cells with TP53RK/Birc5 KO/KD or inhibition, renal tubulointerstitial injury is indeed suppressed. This finding adds to the evidence in support of the crucial role of epithelial cells in the progression of CKD tubulointerstitial fibrosis. Unlike epithelial cells, interstitial fibroblasts, which compose very small aggregate of numbers in kidney, function to remain the structural integrity of the kidney by producing a certain amount of ECM under physiological condition. However, under chronic injuries, interstitial fibroblasts are activated and prompted to proliferation, producing excessive ECM in the interstitial. It is estimated that resident fibroblasts account for 50% of the ECM producing myofibroblast cells in renal fibrosis.^[^ ^35^ ^]^ Thus, by repressing the activation and proliferation of renal resident fibroblasts, TP53RK/Birc5 inhibition retarded kidney fibrosis. The similar pro–fibrotic effect of TP53RK/Birc5 in two types of cells explains the experimental finding that global TP53RK or Birc5 inhibition with high‐throughput tail vein plasmid delivery of CRISPR‐Cas9 plasmids showed a powerful antifibrotic effect.

TP53RK was first cloned from an interleukin‐2‐activated cytotoxic T‐cell subtraction library and described as a p53 interacting protein that is able to phosphorylate p53 at Ser15.^[^ ^6^ ^]^ Being highly expressed in multiple myeloma, colon cancer, and skin cancer, TP53RK is found to promote the proliferation and metastasis of cancer cells. Hence, TP53RK is emerging as a novel oncogene protein and therapeutic target against these cancers.^[^ ^11^, ^36^, ^37^, ^38^ ^]^ Meanwhile, as an important component of the EKC/KEOPS complex that plays a central role in the synthesis of an essential tRNA modification, mutations of TP53RK cause GAMOS.^[^ ^9^ ^]^ Our current research showed that TP53RK also functions to promote CKD kidney fibrosis and the profibrotic effect of TP53RK may rely on its regulation on Birc5, rather than p53.

Birc5 is a multitasking protein that has dual roles in promoting cell proliferation and preventing apoptosis. As with TP53RK, Birc5 overexpression is also well recognized to be associated with tumorigenesis in many types of cancers.^[^ ^39^, ^40^, ^41^, ^42^ ^]^ Meanwhile, Birc5 downregulation is associated with clinical pathology of cystic kidneys and vascular aneurysms.^[^ ^43^ ^]^ A functional study in the kidney has indicated that Birc5 expression in renal proximal tubules is vital for functional and structural recovery of the kidney after ischemia reperfusion.^[^ ^44^ ^]^ Another study reported that Birc5 in urine could be a potential biomarker in children with CKD.^[^ ^45^ ^]^ Our results indicated that TP53RK phosphorylated Birc5 at Thr34 and thus strengthened Birc5 protein stability under the context of kidney fibrosis. Then, we found Birc5 knockdown inhibited while Birc5 overexpression activated PI3K/Akt and MAPK signaling pathways in both cultured kidney epithelial cells and fibroblasts. Strikingly, knocking down of Birc5 also blocked the activation of the two vital signaling pathways in fibrotic kidneys. These data suggested that PI3K/Akt and MAPK pathways, which have been reported to play key roles in promoting fibrosis in kidney,^[^ ^20^ ^]^ could be the downstream signaling of Birc5 in these experimental settings. It has been reported that during mitosis, Birc5 is increasingly expressed in the nucleus, where it binds with Borealin and INCENPa to form the chromosomal passenger complex and aids in chromosomal movement, cytokinesis, and cell proliferation.^[^ ^46^ ^]^ Accordingly, it has also been reported that nuclear, rather than cytoplasmic, localization of Birc5 is a negative prognostic factor for survival in upper urinary tract urothelial carcinoma.^[^ ^47^ ^]^ Combining our research that found p‐Birc5 tends to locate in the nucleus, we might conclude that the phosphorylation form of Birc5 is involved in promoting cell proliferation signaling pathways. However, indirect regulators between Birc5 (p‐Birc5) and PI3K/Akt and MAPK pathways are still possibly existed, which warrant further investigation.

It should be noted that there were some limitations in the present study. First, although activated renal tubular epithelial cells and renal fibroblasts account for the progression of kidney fibrosis in CKD, the current study cannot rule out the possible contributions of immune cells, pericytes, and other cells that are also reported to be involved in this process. Second, it would be better to generate renal tubular and fibroblast specific Birc5 knockout mice to fully demonstrate the function of Birc5 as the downstream signaling of TP53RK in renal fibrosis. Third, UUO and UIR models largely have no effect on BUN and sCr levels since the compensation of the contralateral healthy kidney (Figure S11, Supporting Information). Thus, further studies will be carried out using more cell‐specific transgenic animals and CKD models to address these questions.

## 4 Experimental Section

### Human Kidney Transcriptome Datasets

Data set of patients diagnosed with various forms of CKD was extracted from GEO (https://www.ncbi.nlm.nih.gov/geo, accession: GSE66494). The data set comprised RNA expression profiles of eight normal kidneys and 53 CKD tissues.

### Human Specimens

Pediatric CKD was diagnosed according to KDIGO 2012 Clinical Practice Guideline for the Evaluation and Management of Chronic Kidney Disease.^[^ ^48^ ^]^ Eighteen CKD patients verified by renal biopsy from the Department of Nephrology, Children's Hospital of Nanjing Medical University, were enrolled for the human specimen study. Five normal renal tissues were collected from age and sex‐matched patients without proteinuria who received a partial nephrectomy of a benign renal tumor. The interstitial fibrosis score was evaluated by a blinded pathologist by estimating the extent of tubular atrophy, infiltration of inflammatory cells, and interstitial fibrosis (0, None; 1, ≤25%; 2, 25–50%; 3, ≥50% for each aspect). Individuals with an interstitial fibrosis score of 1–3 were classified as mild, 4–6 as moderate, and those between 7 and 9 as severe fibrosis. Clinical parameters of patients included are shown in Table S1 (Supporting Information). The protocol for the use of renal biopsy samples and nephrectomized tissues from patients was approved by the local committee on human subjects at the Children's Hospital of Nanjing Medical University (project number: 202008089‐1). Written informed consent was provided by each patient.

### Animals

All mice were bred and maintained in a specific pathogen‐free environment maintaining a constant temperature (22±2 °C), an automatic light/dark rhythm (12:12–h light/dark cycle) and free access to water and food. All of the animal experiments were performed in the Animal Core Facility of Nanjing Medical University and all procedures were approved by the Nanjing Medical University Institutional Animal Care and Use Committee (project number: 2007001).

Generation of renal proximal tubular or fibroblast TP53RK conditional knockout mouse strains: First, TP53RK^fl/fl^ mice with a genetic background of C57BL/6J were generated using CRISPR/Cas9 genome engineering by GemPharmatech (Nanjing, Jiangsu, China). Briefly, mutations were generated by co‐injection of Cas9 mRNA and individual gRNAs into one‐cell mouse embryos. sgRNA then directed Cas9 endonuclease cleavage upstream of the predicted promoter of *TP53RK* and between intron 1 and 2 and resulted in loxP site insertions to generate TP53RK^fl/wt^ mice. TP53RK^fl/fl^ mice were then generated by TP53RK^fl/wt^ self‐crossing. TP53RK^fl/fl^ mice were crossed with Kap‐Cre (The Jackson Laboratory, stock No: 008781) and S100A4‐Cre tool mice (The Jackson Laboratory, stock No: 030644) to generate F1. F1 were then crossed with TP53RK^fl/fl^ to generate renal proximal tubular and fibroblast‐specific conditional TP53RK knockout mice (TP53RK^fl/fl^; Kap‐Cre and TP53RK^fl/fl^; S100A‐Cre mice). TP53RK^fl/fl^ mice were used as controls. Genotyping of tail DNA preparations with PCR was performed 1 week after birth.

UUO model: The UUO groups of mice were anesthetized with isoflurane inhalation, and the left ureter was double ligated by 4‐0 silk, while the ureter of the sham group was manipulated without ligation. After 7 or 14 days of modeling, all left renal tissues were collected for further analysis.

UIR model: UIR was induced by clamping the right renal pedicle for 45 min and then released to allow reperfusion, leaving the left kidney intact. The sham group was manipulated without clamping. The mice were in the supine position on a thermostatic pad (37 °C) throughout the whole process.

Pharmaceutical treatment: C57BL/6J mice (7‐week‐old, male) were purchased from GemPharmatech (Nanjing, China) and were allowed to acclimate to the housing environment for 1 week. FA (Cat #HY‐B1350A, MedChemExpress, Princeton, NJ, USA) and YM‐155 (Cat #S1130, Selleck Chemicals, Houston, TX, USA) were dissolved in DMSO and stocked at −80 °C. For FA treatment, the mice were pretreated with FA at 16 mg kg^−1^ d^−1^ via intraperitoneal (i.p.) injection for 10 continuous days before UUO surgery.^[^ ^11^ ^]^ Then the mice were treated daily for seven consecutive days and sacrificed 2 h after the final injection. For YM‐155 treatment, the mice were pretreated with YM‐155 at 3 mg kg^−1^ d^−1^ (i.p) 24 h and 2 h before UUO surgery. Then the mice were treated daily for 14 consecutive days and sacrificed 2 h after the final injection.

### Immunohistochemistry

Human kidney specimens and mice kidney sections were fixed with 4% paraformaldehyde (PFA) for over 24 h and then were embedded in wax before being sliced into 3‐µm sections. After dewaxed gradually with xylene and ethanol, the tissue slides were microwaved in citrate antigen repair solution (Cat #P0083, Beyotime Biotechnology, Shanghai, China) for 10 min and then treated with 3% H_2_O_2_ for 10 min to block the endogenous peroxidase activity. Subsequently, the tissue slides were blocked (Cat #P0260, Beyotime Biotechnology) for 1 h at room temperature and then incubated with primary antibodies: anti‐TP53RK (Cat #ab279377, Abcam, Cambridge, MA, USA) and anti‐Birc5 (Cat #ab469, Abcam) overnight at 4 °C. The next day, horseradish peroxidase was applied. Localization of peroxidase conjugates was determined using a 3, 3'‐diaminobenzidine kit (Cat #PV‐9000, Zsbio, Beijing, China) and nuclei were stained with hematoxylin. Images of tissue slides were collected with an Olympus BX51 microscope (Olympus, Tokyo, Japan) and the intensity of the positive staining area was quantified with the Image‐Pro Plus software (Media Cybernetics Inc., Rockville, MD, USA).

### Masson Trichrome Staining

Masson trichrome staining was carried out using a commercial kit (Cat #g1006, Servicebio, Wuhan, China) according to the manufacturer's instructions. Briefly, deparaffinized tissues were incubated overnight with solution A then placed at 65 °C for 30 min. The nuclei were stained for 1 min by a mixture of B and C, and then differentiated by 1% hydrochloric acid alcohol. The tissues were then treated with phosphomolybdic acid (E solution) for 1 min. Thereafter, the muscle fibers of the tissues were dyed red with Ponceau (D solution) for 8 min, while the collagen fibers were dyed blue with aniline blue (F solution) for 8–30 s. The two colors could be distinguished better after differentiation with 1% glacial acetic acid. The pathological images were collected using Olympus BX51 microscope, and quantification of the fibrosis area was analyzed by Image‐Pro Plus software.

### Cell Culture, Transfection, and Treatment

mPTC, HK2, and NRK‐49F cells were all obtained from the American Type Culture Collection (Manassas, VA, USA). The mPTCs were cultured in a Dulbecco's modified Eagle medium (DMEM)/F12 medium (Cat #C11330500BT, Gibco, Thermo Fisher Scientific, Halethorpe, MD); HK2 and NRK‐49Fs were cultured in DMEM (Cat #C11995500BT, Gibco) supplemented with 10% (v/v) fetal bovine serum (Cat # 10099–141C, Gibco) and 1% penicillin/streptomycin solution in a humidified incubator supplemented with 5% CO_2_ at 37 °C. Cells were stimulated with indicated concentrations of TGF‐*β*1 (Cat #240‐B, R&D systems, Minneapolis, MN, USA) for 24 h.

### Knockdown of TP53RK and Birc5 Expression

The CRISPR/Cas9 technique was used for genetic knockdown in mPTCs and NRK‐49F cells. The sgRNA sequences were as follows: 5’‐CCGGGTGCCGGTAACTCTTC‐3’ and 5’‐AAAGGCTTCAAACGCGGTCT‐3’ for mouse TP53RK; 5’‐TGACGAGGACCTCATTCACG‐3’ and 5’‐GTCCACAAAGAAGACGACGG‐3’ for rat TP53RK; 5’‐AGTTCTTGAAGGTGGCGATG‐3’ and 5’‐AATCAGGCTCGTTCTCGGTA‐3’for mouse Birc5; 5’‐CTACGGCGCTGCCCCCGATC‐3’ and 5’‐TGTAGATCCGGTGGTCCTTA‐3’for rat Birc5. The sgRNAs were phosphorylated, annealed into double chains, and cloned into the vector pSpCas9 (BB)‐2A‐Puro (PX459) v2.0 (Cat #62988, Addgene plasmid). The constructed plasmid or empty vector was transfected into cells using PolyJet In Vitro DNA Transfection Reagent (Cat #SL100688, SignaGen Laboratories, USA) according to the manufacturer's protocol.

### Pharmaceutical Inhibition of TP53RK and Birc5

Cells were preincubated with 5 × 10^−6^, 10 × 10^−6^, or 20 × 10^−6^ m FA or 1 × 10^−9^, 2.5 × 10^−9^, or 5 × 10^−9^ m (or 1 × 10^−9^, 2 × 10^−9^, 3 × 10^−9^ m) YM‐155 for 2 h before TGF‐*β*1 stimulation. mPTCs were stimulated with TGF‐*β*1 at a concentration of 10 ng mL^−1^ and NRK‐49Fs were treated with TGF‐*β*1 at a concentration of 5 ng mL^−1^ for 24 h.

### Evaluation on Birc5 Protein Stability

The mPTCs were first transfected with TP53RK overexpression plasmid or vector using PolyJet In Vitro DNA Transfection Reagent (Cat #SL100688, SignaGen Laboratories). Then, cells were treated with CHX (Cat #S7418, Selleck Chemicals) at a concentration of 10 µg mL^−1^ and collected at 0, 3, 6, 9, and 12 h. Time course protein levels of Birc5 were monitored with Western blot.

### Western Blot Analysis

Tissue and cell proteins were extracted with RIPA buffer (Cat #P0013B, Beyotime Biotechnology) containing protease inhibitors (Cat #04693132001, Roche, Laval, Canada). Concentration of the extracted protein was measured with a BCA protein assay kit (Cat #P0010, Beyotime Biotechnology). Protein extracts were then mixed with 5 × SDS buffer (Cat #P0015, Beyotime Biotechnology), boiled for 10 min, and stored at –80 °C. The extracted protein samples (30–50 µg) were subjected to SDS–PAGE, and then transferred onto polyvinylidene fluoride membranes (Bio‐Rad, CA, USA). Afterwards, the membranes were blocked at room temperature for 1 h and incubated with primary antibodies overnight at 4 °C, followed by incubation with HRP‐conjugated secondary antibodies for 1 h the next day. Immunoblotted bands were ultimately detected with chemiluminescence reagent (Cat #P0018S, Beyotime Biotechnology) on a ChemiDoc XRS+ System (Bio‐Rad). The following antibodies were commercially obtained for Western blot analysis: anti‐TP53RK (1:200, Cat #ab37606, Abcam or 1:500, Cat #A14952, Abclonal, Wuhan, China); anti‐Birc5 (1:500, Cat #10508‐1‐AP, ProteinTech Group); anti‐p‐Birc5 (phospho Thr34, 1:500, Cat #ab138653, Abcam); anti‐FN (1:1000, Cat #ab2413, Abcam); anti‐VIM (1:2000, Cat #ab92547, Abcam); anti‐POSTN (1:500, Cat #NBP1‐30042, Novus Biologicals, Littleton, Colorado, USA); anti‐*α*‐SMA (1:1000, Cat #14395‐1‐AP, Proteintech Group, Rosemont, IL, USA); anti‐p53 (1:1000, Cat #2524S, Cell Signaling Technology, Danvers, MA, USA); anti‐p‐p53 (phospho Ser10, 1:1000, Cat #9284S, Cell Signaling Technology); anti‐GAPDH (1:1000, Cat #60004‐1‐Ig, Proteintech Group); anti‐*β*‐Actin (1:2000, Cat #60009‐1‐Ig, Proteintech Group); HRP‐labeled goat anti‐mouse secondary antibody (1:2000, Cat #A0216, Beyotime Biotechnology) and HRP‐labeled goat anti‐rabbit secondary antibody (1:2000, Cat #A0208, Beyotime Biotechnology).

### qRT‐PCR

Total tissue and cell RNAs were extracted using the RNAiso Plus reagent (Cat #9108, Takara Biotechnology, Dalian, China) according to the manufacturer's protocol. RNAs were redissolved with diethypyrocarbonate (DEPC) treated water (Cat #R0021, Beyotime Biotechnology) and reverse transcribed to cDNA using PrimeScript RT reagent Kit (Cat #RR037A, Takara Biotechnology). qRT‐PCR was performed using SYBR Select Master Mix (Cat #q111‐02/03, Vazyme, Nanjing, China) on a QuantStudio 3 Real‐Time PCR system (Applied Biosystems, Foster City, CA, USA) following the cycling program: preliminary denaturation 95 °C for 10 min, followed by repeats of 95 °C for 15 s and 60 °C for 1 min. Relative gene expression of mRNA was normalized to *Gapdh* and calculated using the 2^−△△Ct^ method.

### Immunofluorescence Staining

HK‐2 cells were cultured in glass‐bottom cell culture dishes (Cat #801001, NEST, Nantong, Jiangsu, China) and stimulated with TGF‐*β*1 (10 ng mL^−1^). After several washes with PBS, cells were then fixed with 4% PFA for 30 min at room temperature and permeabilized with 0.1% Triton X‐100/PBS for 5 min. Tissue slides were dewaxed, repaired, and treated with 3% H_2_O_2_ as described above. Then, cells and tissue slides were blocked with 2% BSA/PBS for 1 h, incubated with primary antibodies overnight at 4 °C. The following antibodies were commercially obtained for immunofluorescence staining: anti‐TP53RK (1:40, Cat#MA5‐22905, Invitrogen, Carlsbad, CA, USA), anti‐Birc5 (1:200, Cat #ab469, Abcam), anti‐p‐Birc5 (1:50, Cat #PA1‐16853, Invitrogen), anti‐Ki‐67 (1:200, Cat #9129T, Cell Signaling Technology), anti‐S100A4 (1:50, Cat #A19109, Abclonal or 1:20, Cat #66489‐1‐Ig, ProteinTech Group). Proximal tubule was labeled with fluorescein‐labeled LTL (1:100, Cat #FL‐1321‐2, Vector laboratories, Burlingame, CA, USA) and nucleus was labeled with DAPI. The Alexa Fluor 488‐conjugated goat anti‐rabbit IgG (1:500, Cat #ab150077, Abcam) and Alexa Fluor 594‐conjugated goat anti‐mouse IgG (1:500, Cat #A‐11037, Thermofisher Scientific, Waltham, MA, USA) secondary antibodies were applied after washing with PBS/T for three times. Then cells and tissue slides were viewed and imaged under a Zeiss 710 confocal microscopy (Zeiss, Jena, Germany) or an Olympus BX51 microscope (Olympus).

*Co‐IP*: mPTCs and NRK‐49Fs were transfected with TP53RK‐Flag and Birc5‐His plasmids or vector and collected 24 h after transfection. Cells were lysed with NP40 lysis buffer (Cat #P0013F, Beyotime Biotechnology) containing 1% protease inhibitor cocktail (Cat #04693132001, Roche). Whole cell lysate was immunoprecipitated with antibody together with protein A/G magnetic beads (Cat #B23201, Bimake, Shanghai, China). The antibodies used for immunoprecipitating were anti‐His (Cat #12698S, Cell Signaling Technology), anti‐Flag (Cat #F3165, Sigma–Aldrich, St Louis, MO, USA), normal mouse IgG (Cat #sc‐2025, Santa Cruz Biotechnology, Santa Cruz, CA, USA), and normal rabbit IgG (Cat #2729S, Cell Signaling Technology). The immunoprecipitates were eluted with 1 × SDS Loading Buffer (Cat #P0015, Beyotime Biotechnology), separated by SDS–PAGE and analyzed by Western blotting.

### EdU Cell Proliferation Assay

For the EdU cell proliferation assay, approximately 1 × 10^5^ cells per well were seeded and cultured in 12‐well plates. After indicated treatments, NRK‐49Fs were incubated with EdU solution (Cat#C10338, RiboBio, Guangzhou, China) for 2 h and then stained with Hoechst 33342. Images were collected by an Olympus IX73 microscopy (Olympus). The EdU incorporation rate was quantified by normalizing the number of EdU positive cells against total nucleus number. At least 800 cells were viewed and counted in each well.

### Statistical Analysis

Data are expressed as the mean ± SEM. Statistical significance between two groups was determined with two‐tailed unpaired Student's *t* test. When more than two groups were compared, one‐way ANOVA followed by Dunnett's multiple comparison test or two‐way ANOVA followed by Tukey's multiple comparison test was used to determined differences between two groups of interest. *p* < 0.05 was considered statistically significant.

## Conflict of Interest

The authors declare no conflict of interest.

## Author Contributions

M.W. and Q.J. contributed equally to this work. Z.J, A.Z., Y. Z., and M.W. performed conceptualization. M.W., Q.J., X.X., J.F., W.C., M.M, R.G., S.Z., and Y.G. performed methodology. M.W., Q.J., X.X., J.F., and W.C. performed investigation. M.W., Q.J., X.X., and J.F. performed visualization. A.Z., Z.J, Y.Z., S.H., and M.W. performed funding acquisition. A.Z., Z.J, Y.Z., and S.H. performed project administration. A.Z., Z.J., Y.Z., and S.H. performed supervision. M.W. and Q.J. wrote the original draft. Z.J, A.Z., and M.W. reviewed and edited the work.

## Supporting information

Supporting Information

## Data Availability

The data that support the findings of this study are available from the corresponding author upon reasonable request.
