# Supplementary material for: TP53RK Drives the Progression of Chronic Kidney Disease by Phosphorylating Birc5
Source: Adv Sci (Weinh). 2023 Jun 29;10(25):2301753. doi: 10.1002/advs.202301753 (PMC10477881; doi:10.1002/advs.202301753)
Supplement: Supplementary file 1 — Supporting Information [file ADVS-10-2301753-s001.pdf]

ADVANCED SCIENCE
Open Access

Supporting Information

for *Adv. Sci.*, DOI 10.1002/advs.202301753

TP53RK Drives the Progression of Chronic Kidney Disease by Phosphorylating Birc5

Mengqiu Wu, Qianqian Jin, Xinyue Xu, Jiaojiao Fan, Weiyi Chen, Mengqiu Miao, Ran Gu, Shengnan Zhang, Yan Guo, Songming Huang, Yue Zhang, Aihua Zhang* and Zhanjun Jia**

TP53RK drives the progression of chronic kidney disease by phosphorylating Birc5

Mengqiu Wu, Qianqian Jin, Xinyue Xu, Jiaojiao Fan, Weiyi Chen, Mengqiu Miao, Ran Gu, Shengnan Zhang, Yan Guo, Songming Huang, Yue Zhang, Aihua Zhang*, Zhanjun Jia**

M. Wu, Q. Jin, M. Miao, R. Gu, S. Zhang, Y. Guo, S. Huang, Y. Zhang, A. Zhang, Z. Jia

Department of Nephrology

Nanjing Key Laboratory of Pediatrics

Jiangsu Key Laboratory of Pediatrics

Children's Hospital of Nanjing Medical University

Nanjing Medical University

Nanjing 210008, China

Email: jiazj72@hotmail.com; zhaihua@njmu.edu.cn; zyflora2006@hotmail.com

X. Xu, J. Fan

School of Medicine, Southeast University

Nanjing 210009, China

W. Chen

Department of Emergency Medicine, Children's Hospital of Nanjing Medical University

Nanjing 210008, China

Keywords: chronic kidney disease, fibrosis, TP53RK, Birc5, phosphorylation, renal tubular cells, renal fibroblasts

Supplemental Materials

Experimental Section

Supplemental Figures

Table S1

Experimental Section

RNA sequencing (RNA-Seq): UIR and sham operated mice were sacrificed at 1, 3, and 21 days after surgery. Kidney tissues were harvested and frozen rapidly in liquid nitrogen. RNA isolation, library construction, and sequencing were performed by Beijing Genomic Institution using a BGISEQ-500 RNA-seq platform (Beijing Genomic Institution, Shenzhen, China). The mouse GRCm38.p5 reference genome was used to map the clean tags.

High-throughput tail vein plasmid delivery: Wild-type C57BL/6J mice (7 weeks old, male) were purchased from GemPharmatech (Nanjing, Jiangsu, China) and were allowed to acclimate to the housing environment for a week. TP53RK overexpression, TP53RK and Birc5 targeted CRISPR/Cas9 plasmids, as well as the corresponding vectors were dissolved in saline to a working concentration of 30 μg/mL before injection. Plasmids amounting to 2 mL were delivered to the tail vein of mice within 10 s. UUO or sham operation was performed 36 h after injection. Animal experiments were performed in the Animal Core Facility of Nanjing Medical University and all of the procedures were approved by the Nanjing Medical University Institutional Animal Care and Use Committee.

Hematoxylin and eosin (H&E) staining: H&E staining was carried out using a commercial kit (Cat #G1120, Solarbio, Wuhan, China) according to the manufacturer's instructions. Briefly, the tissue

slides were stained with hematoxylin solution for 10 min, differentiated with differentiation solution for 3 min and re-dyed with eosin Y aqueous solution for 1 min. The tissue slides were viewed and imaged under a Olympus BX51 microscope (Olympus, Tokyo, Japan).

Pharmaceutical combination treatment of UUO mice: To explore the combinational effect of fusidic acid (FA) and YM-155 treatment in UUO mice, 8 weeks old male C57BL/6J mice (GemPharmatech, Nanjing, China) were randomly divided into five groups: sham group, UUO group, UUO+FA group, UUO+YM-155 group, and UUO+FA+YM-155 group. FA was given to mice at 10 mg/kg/d and YM-155 was employed at 3 mg/kg/d, via intraperitoneal injection 24 h and 2 h before UUO surgery. Then the mice were treated daily for 7 consecutive days and sacrificed 2 h after the final injection.

Supplemental Figures

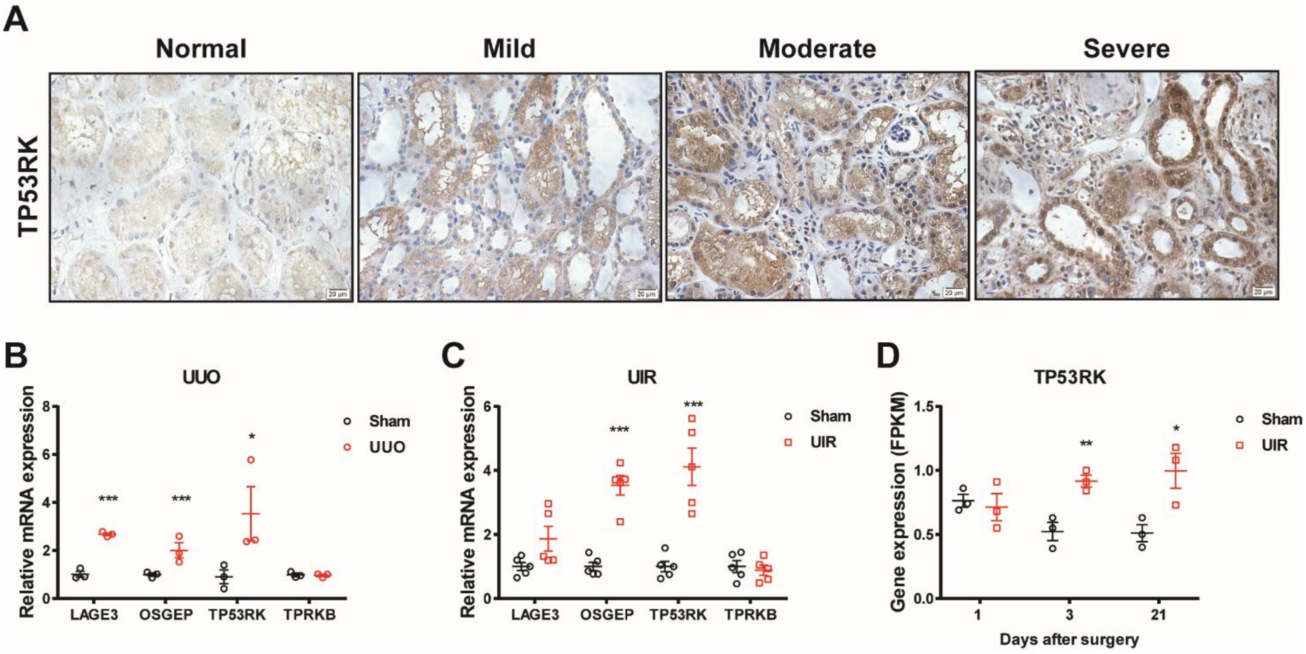

Figure S1 Expression pattern of TP53RK in fibrotic kidneys

(A) Representative immunohistochemical staining of TP53RK in normal group (n = 5) and in hospitalized CKD patients with mild (n = 5), moderate (n = 7), and severe (n = 6) fibrosis. (B–C) qRT-PCR analysis of the four main subunits compromising the EKC/KEOPS complex (LAGE3, OSGEP, TP53RK and TPRKB) in UUO (day 7) (n = 3) and UIR (day 21) (n = 5) kidneys. (D) RNA-Seq analysis of TP53RK in sham and UIR kidneys 1, 3, and 21 days after surgery (n = 3). Expression of genes was measured using fragments per kilobase of transcript per million mapped reads (FPKM). Data are presented as mean ± SEM; two-tailed unpaired t-test was used to determine statistical significance; $^{*}p < 0.05$, $^{**}p < 0.01$ and $^{***}p < 0.001$ compared with the sham group.

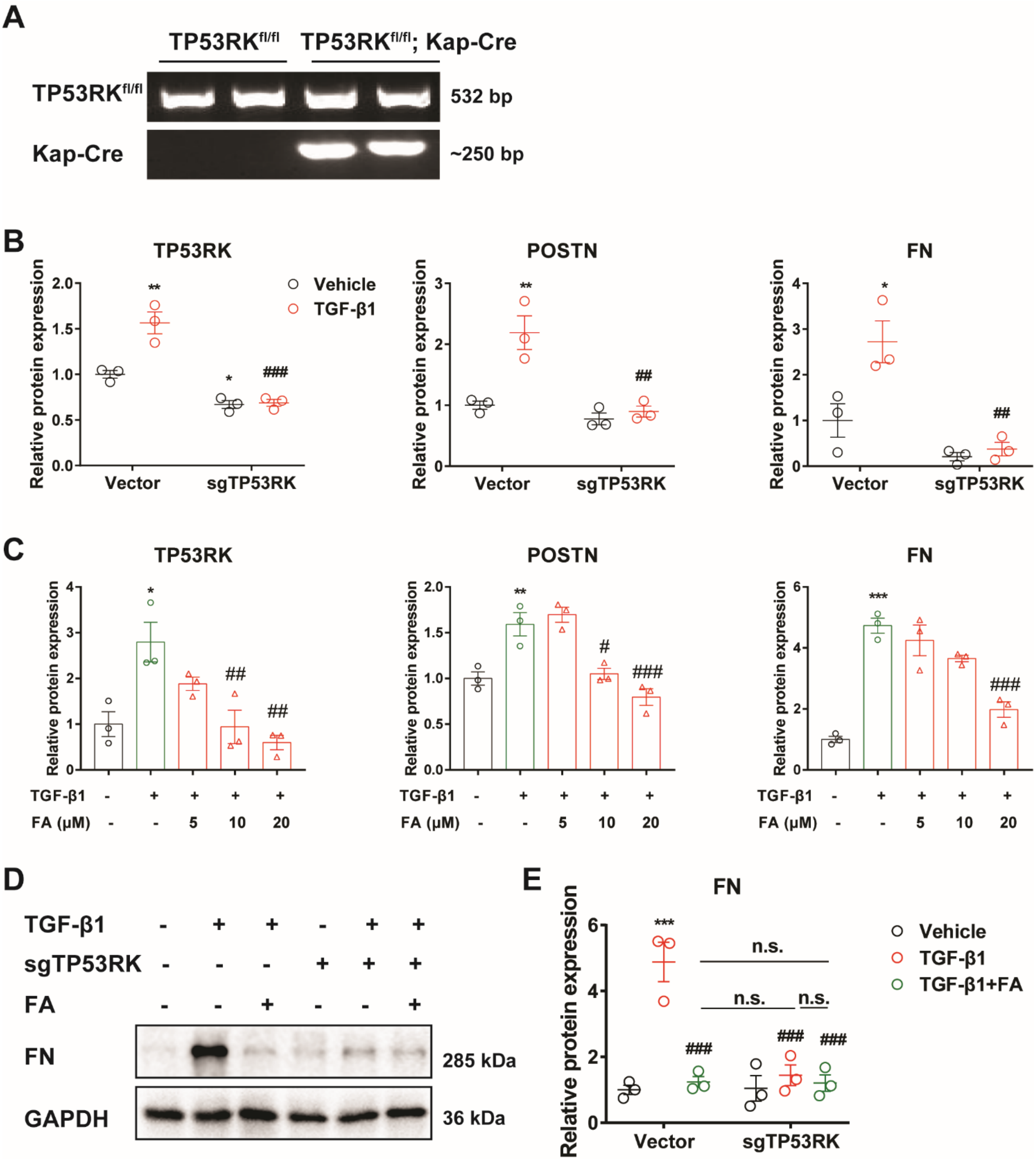

Figure S2 Genetic or pharmaceutic inhibition of TP53RK alleviated TGF-β1-induced p-EMT in cultured mouse kidney epithelial cells

(A) Genotyping of tail DNA preparations with PCR. (B) Control (vector) and TP53RK KD (sgTP53RK) mPTCs were treated with TGF-β1 (10 ng/mL) for 24 h and harvested for western blot analysis of TP53RK, POSTN, and FN.

Protein semi-quantification is presented ($n = 3$). (C) mPTCs were pre-treated with vehicle or fusidic acid (FA) (5, 10, or 20 μM) 2 h before TGF-β1 (10 ng/mL) treatment for another 24 h. Cells were harvested for western blot analysis of TP53RK, POSTN, and FN. Protein semi-quantification is presented ($n = 3$). (D–E) Control (vector) or TP53RK KD (sgTP53RK) mPTCs were pre-treated with vehicle or FA (10 μM) 2 h before TGF-β1 (10 ng/mL) treatment for another 24 h. Representative western blot and semi-quantification of FN in each group were shown ($n = 3$). Data are presented as mean ± SEM; two-way ANOVA followed by Tukey's multiple comparisons test was used to determine statistical significance of (B) and (E); one-way ANOVA followed by Dunnett's multiple comparisons test was used to determine statistical significance of (C); $^{*}p < 0.05$ and $^{***}p < 0.001$ compared with the Vector+Vehicle or Vehicle group; $^{\#}p < 0.05$ and $^{\#\#}p < 0.01$ and $^{\#\#\#}p < 0.001$ compared with the Vector+TGF-β1 or TGF-β1 group.

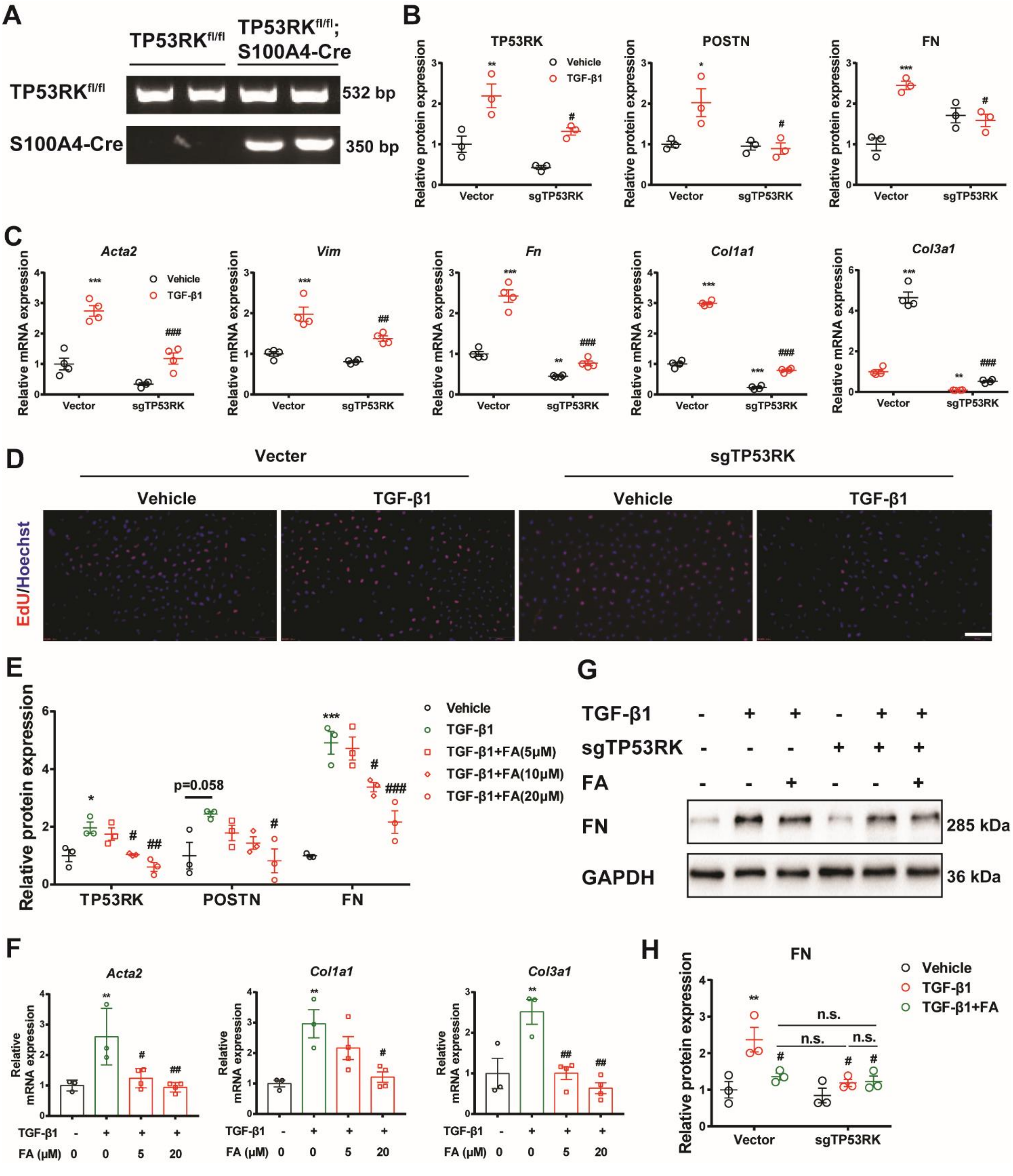

Figure S3 Genetic or pharmaceutic inhibition of TP53RK alleviated TGF-β1-induced proliferation and activation of cultured rat kidney fibroblasts

(A) Genotyping of tail DNA preparations with PCR. (B) Control (vector) and TP53RK KD (sgTP53RK) NRK-49Fs were treated with TGF-β1 (5 ng/mL) for 24 h and harvested for western blot analysis. Semi-quantification of TP53RK,

POSTN, and FN protein expression was presented (n = 3). (C) Control (vector) and TP53RK KD (sgTP53RK) NRK-49Fs were treated as in (B). qRT-PCR analysis of fibrosis markers *Acta2*, *Vim*, *Fn*, *Col1a1* and *Col3a1* was presented (n = 4). The two-way ANOVA was used for differential expression analysis. (D) Control and TP53RK knockdown NRK-49Fs were treated as in (B). Representative images were shown (n = 4). Scale bar: 10 μm. (E) NRK-49F cells were pre-treated with vehicle or FA (5, 10, or 20 μM) 2 h before TGF-β1 (5 ng/mL) treatment for another 24 h and collected for western blot analysis. Semi-quantification of TP53RK, POSTN, and FN was presented (n = 3). (F) NRK-49Fs were pre-treated with vehicle or FA (5 or 20 μM) 2 h before TGF-β1 (5 ng/mL) treatment. Cells were collected for qRT-PCR analysis of *Acta2*, *Col1a1* and *Col3a1* 24 h after TGF-β1 stimuli (n = 3–4). (G–H) Control (vector) or TP53RK KD (sgTP53RK) NRK-49Fs were pre-treated with vehicle or FA (10 μM) 2 h before TGF-β1 (10 ng/mL) treatment for another 24 h. Representative western blot and semi-quantification of FN in each group were shown (n = 3). Data are presented as mean ± SEM; two-way ANOVA followed by Tukey's multiple comparisons test was used to determine statistical significance of (B), (C) and (H); one-way ANOVA followed by Dunnett's multiple comparisons test was used to determine statistical significance of (E–F); $^{*}p < 0.05$, $^{**}p < 0.01$ and $^{***}p < 0.001$ compared with the Vector+Vehicle or Vehicle group; $^{\#}p < 0.05$, $^{\#\#}p < 0.01$ and $^{\#\#\#}p < 0.001$ compared with the Vector+TGF-β1 or TGF-β1 group.

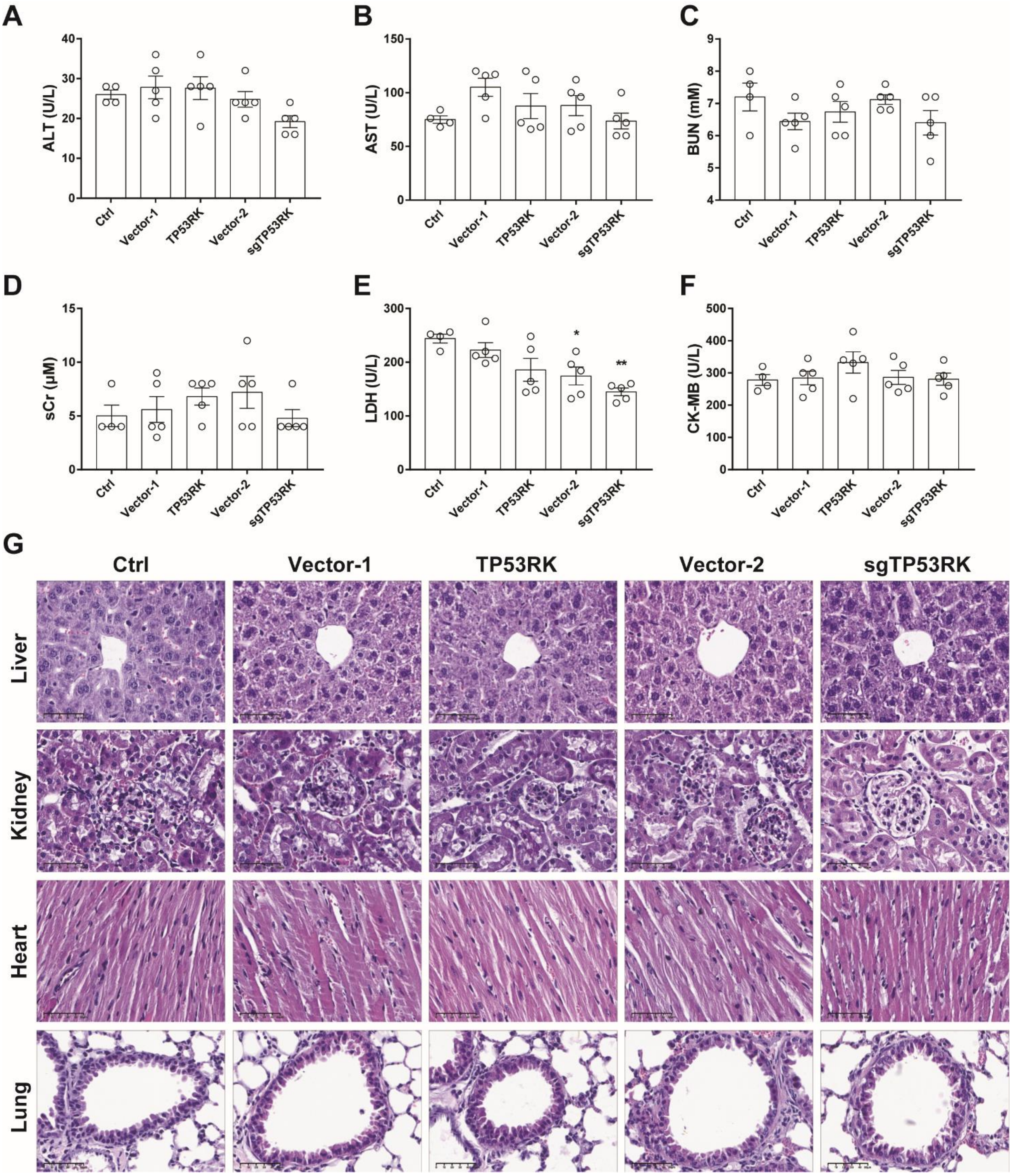

Figure S4 Safety assessment of high-throughput tail vein delivery of TP53RK over-expression and CRISPR/Cas9 plasmids

Mice were randomly divided into five groups. The control (ctrl) group receive tail vein injection of 200 μL saline and the other groups received hydrodynamic-based tail vein plasmid delivery of corresponding plasmids. Mice were

euthanized 7 days after injection. (A) Measurement of serum aspartate aminotransferase (AST), alanine aminotransferase (ALT), blood urea nitrogen (BUN), serum creatinine (sCr), lactic dehydrogenase (LDH) and creatine kinase myocardial band (CK-MB). n=4–5 mice per group. (B) Representative images of H&E staining of liver, kidney, heart and lung. Scale bar, 50 μm. Vector-1 represents empty vector of TP53RKover-expression plasmid; vector-2 represents empty vector of TP53RK targeting CRISPR/Cas9 plasmid. Data are presented as mean ± SEM; one-way ANOVA followed by Dunnett's multiple comparisons test was used to determine statistical significance; $^{*}p < 0.05$ and $^{**}p < 0.01$ compared with the ctrl group.

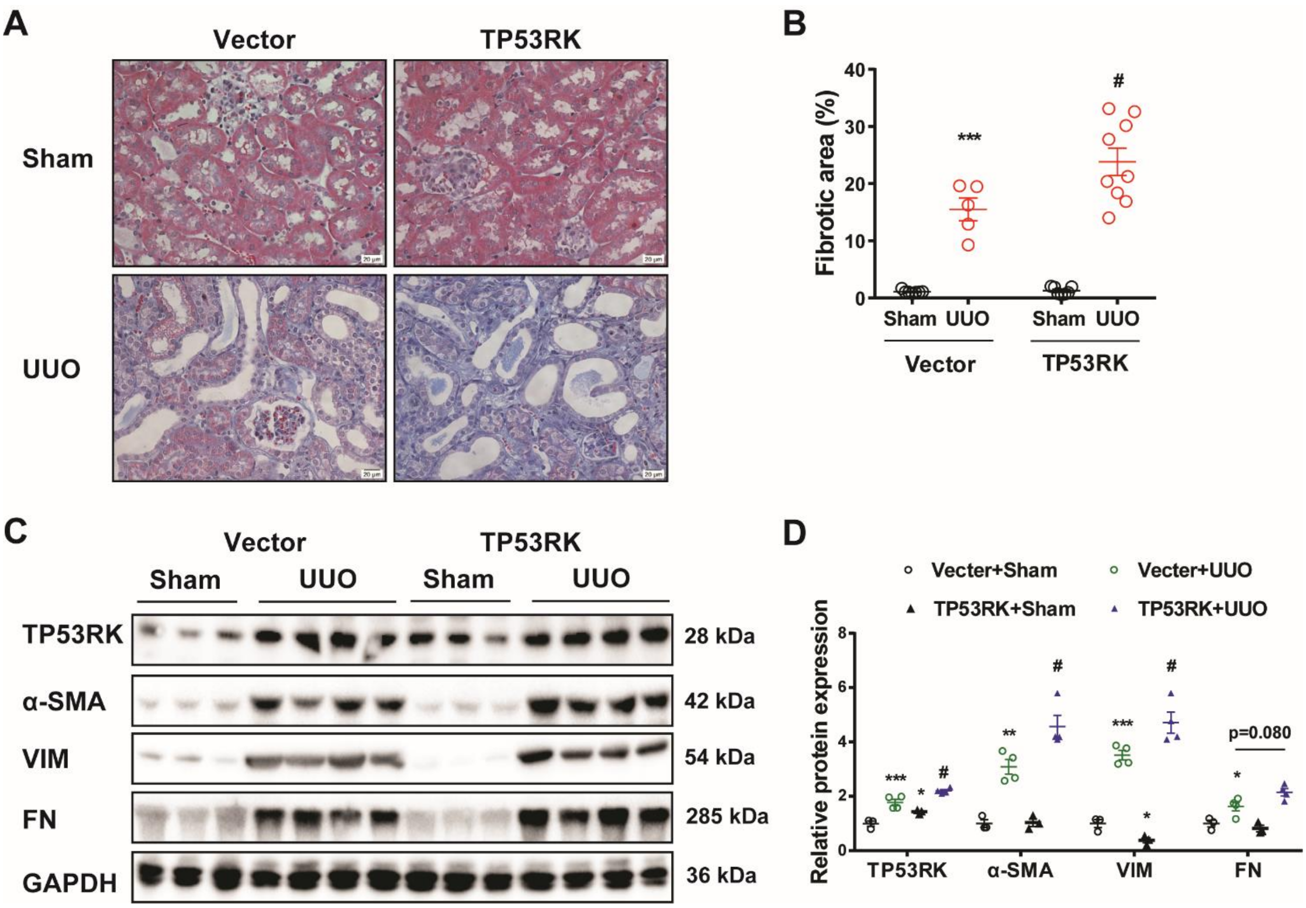

Figure S5 Global overexpression of TP53RK aggravated UUO-induced kidney fibrosis

TP53RK was overexpressed by hydrodynamic-based tail vein plasmid delivery of TP53RK over-expression plasmids. Mice were subjected to sham or UUO surgery 36 h later and euthanized 7 days after establishment of the model. (A–B) Deposition of total fibrosis in kidney tissues was determined by Masson's trichrome staining. Scale bar, 20 μm. Quantification analysis of fibrotic area was shown in parallel (n = 5–9). (C–D) Representative immunoblots and semi-quantification of TP53RK, α-SMA, VIM and FN in each group (n = 3–4). Data are presented as mean ± SEM; two-way ANOVA followed by Tukey's multiple comparisons test was used to determine statistical significance; $^{*}p < 0.05$, $^{**}p < 0.01$ and $^{***}p < 0.001$ compared with the vector+sham group; $^{\#}p < 0.05$ compared with the TP53RK+UUO group.

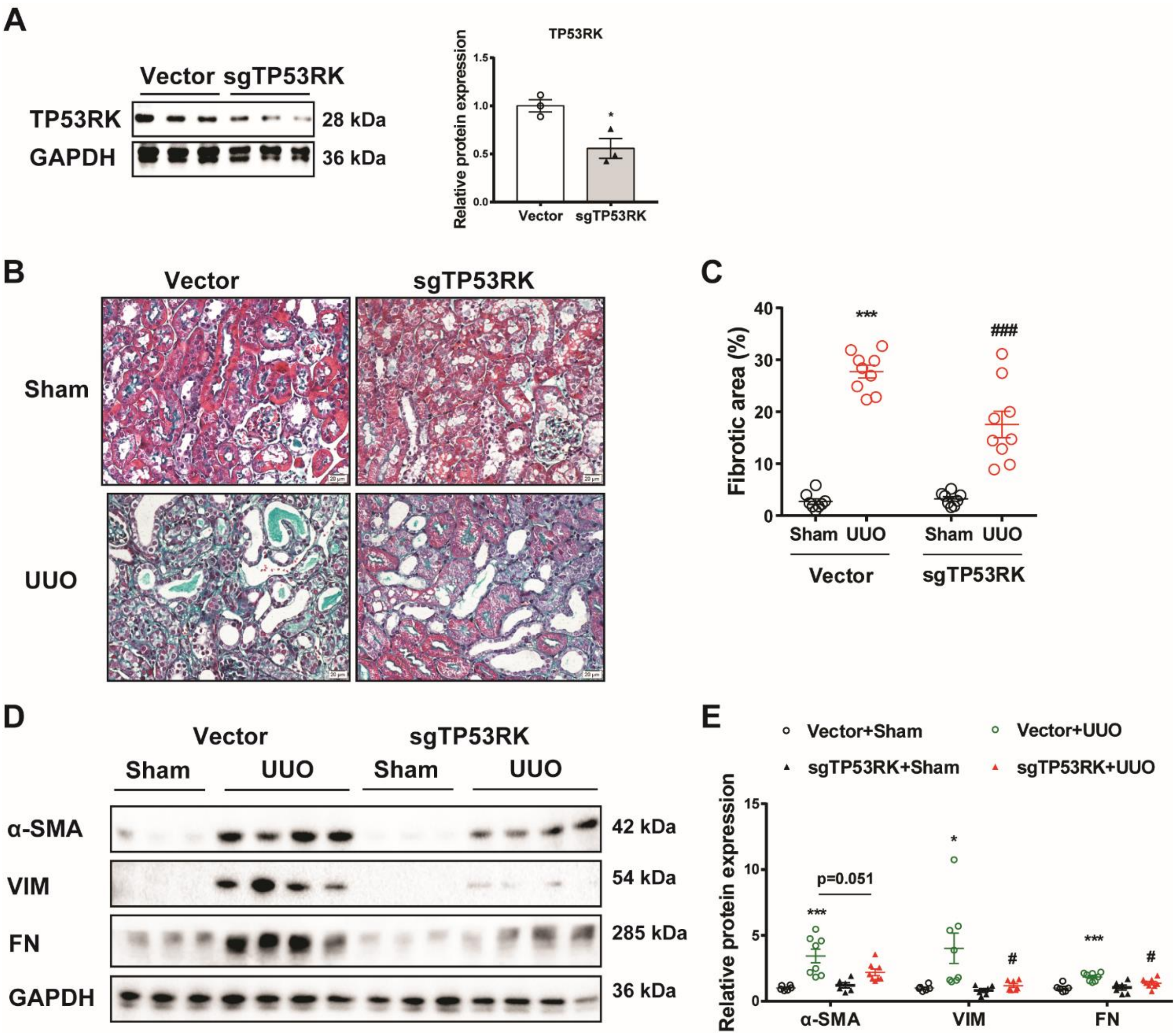

Figure S6 Global TP53RK knockdown with the CRISPR/Cas9 plasmids alleviated UUO-induced kidney fibrosis

(A–B) Mice were subjected to hydrodynamic-based tail vein plasmid delivery of TP53RK targeted CRISPR/Cas9 plasmids (sgTP53RK). TP53RK expression in the kidneys was estimated 72 h after injection (n = 3). (B–C) Thirty-six hours after tail vein injection of sgTP53RK plasmids, mice were subjected to sham or UUO surgery and euthanized 7 days after establishment of the model. Total fibrosis in kidney tissues was determined and quantified by Masson's trichrome staining (n = 8–9). Scale bar, 20 μm. (D–E) Western blot analysis and semi-quantification of α-SMA, VIM, and FN expression in kidneys of each group (n = 6–8). Data are presented as mean ± SEM; two-tailed

unpaired t-test was used to determine statistical significance of (A); two-way ANOVA followed by Tukey's multiple comparisons test was used to determine statistical significance of (C) and (E); $^{*}p < 0.05$, and $^{***}p < 0.001$ compared with the vector or the vector+sham group; $^{\#}p < 0.05$, and $^{\#\#\#}p < 0.001$ compared with the vector+UUO group.

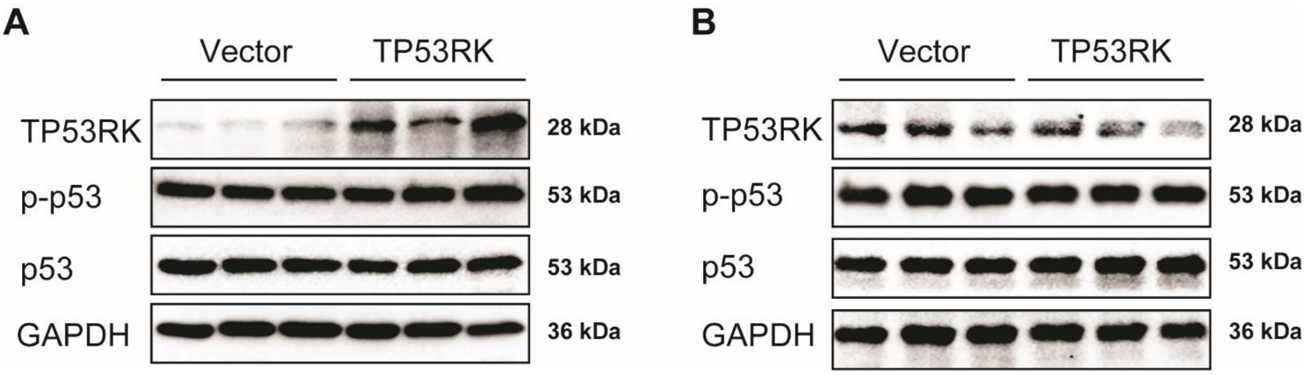

Figure S7 TP53RK may not phosphorylate p53 on Ser15 in kidney epithelial cells

(A) mPTCs were transfected with TP53RK over-expression plasmids or vector. After 24 h, cells were harvested for western blot analysis of TP53RK, p-p53 (Ser15) and total p53 levels (n = 3). (B) mPTCs were transfected with TP53RK targeted CRISPR/Cas9 plasmids (sgTP53RK) or corresponding empty vector. After 24 h, cells were harvested for western blot analysis of TP53RK, p-p53 (Ser15) and total p53 levels (n = 3).

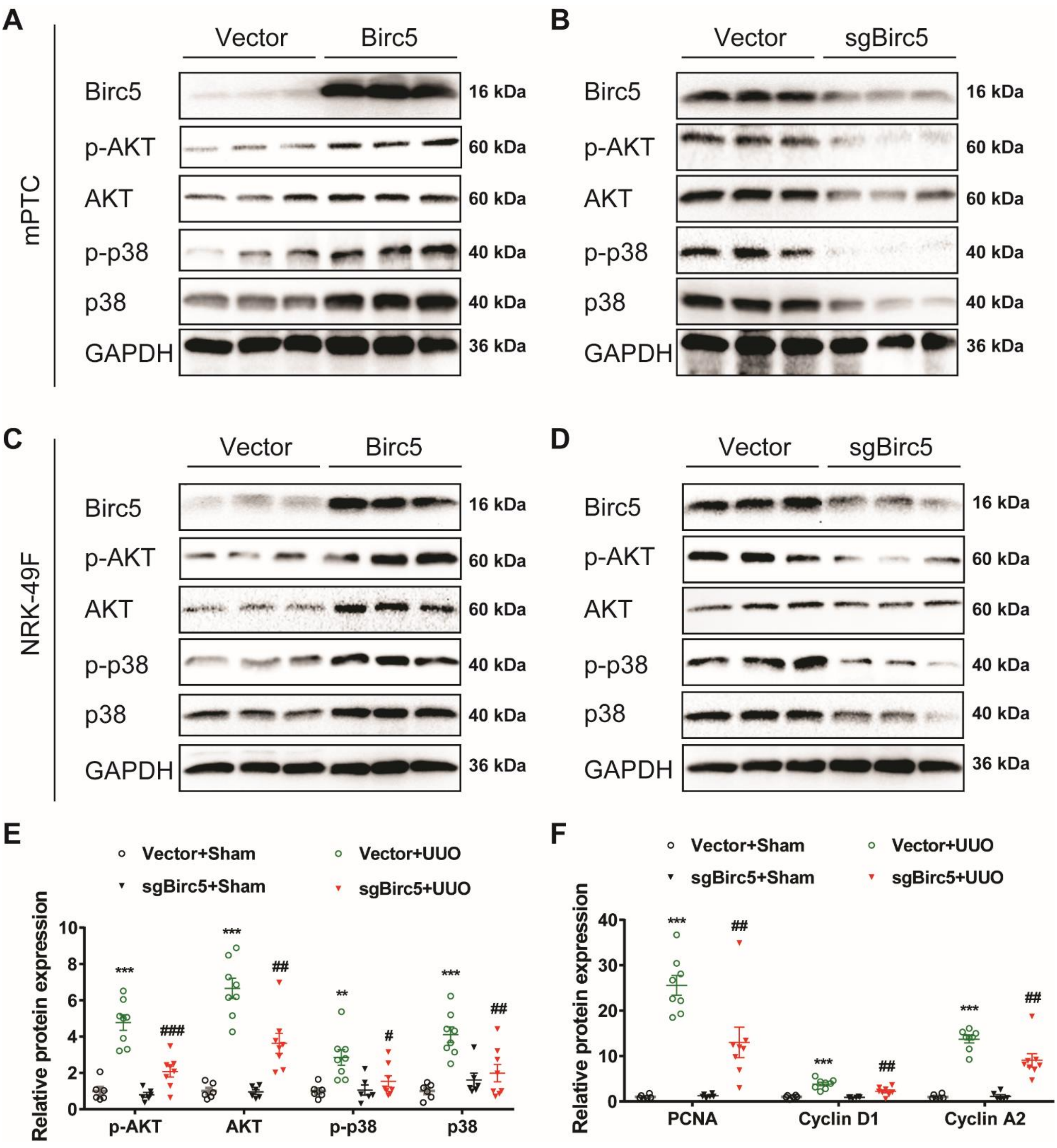

Figure S8 Birc5 knocking down mitigated UUO-induced activation of PI3K/Akt and MAPK signaling pathways

(A–B) mPTCs were transfected with Birc5 over-expression or sgBirc5 plasmids. After 24 h, cells were harvested for western blot analysis of Birc5, p-AKT, AKT, p-p38 and p38 levels (n = 3). (C–D) NRK-49Fs were transfected with Birc5 over-expression or sgBirc5 plasmids. After 24 h, cells were harvested for western blot analysis of Birc5, p-

AKT, AKT, p-p38 and p38 levels (n = 3). (E–F) Mice received high-throughput tail vein delivery of empty vector or Birc5 targeted CRISPR/Cas9 plasmids (sgBirc5) were subjected to UUO or sham operation and euthanized 7 days after establishment of the model. Kidney tissues were harvested for immunoblot analysis of p-AKT, AKT, p-p38 and p38, PCNA, cyclin D1, and cyclin A2 expression. Semi-quantification of the protein expression was shown. Data are presented as mean ± SEM; two-way ANOVA followed by Tukey's multiple comparisons test was used to determine statistical significance; $^{**}p < 0.01$ and $^{***}p < 0.001$ compared with the Vector+Sham group; $^{\#}p < 0.05$, $^{\#\#}p < 0.01$, and $^{\#\#\#}p < 0.001$ compared with the Vector+UUO group.

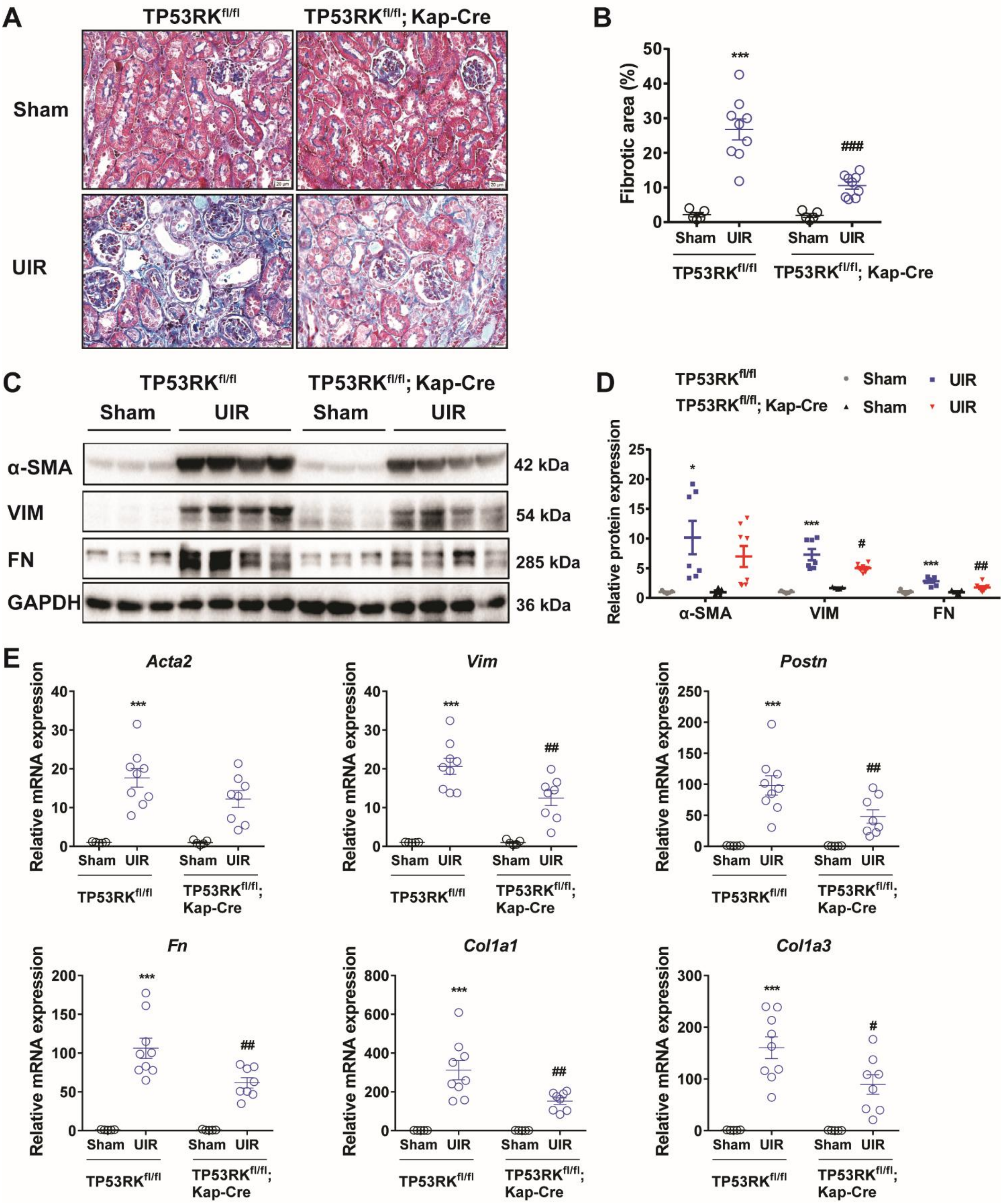

Figure S9 Tubular conditional knockout of TP53RK attenuated AKI–CKD transition

TP53RK$^{fl/fl}$ and TP53RK$^{fl/fl}$; Kap-Cre mice were subjected to 45-min UIR and euthanized at day 21. (A–B) Representative images of Masson trichrome staining of kidney tissues (A) and quantification of fibrotic area (B) in

corresponding groups (n = 5–9). Scale bar, 20 μm. (C–D) Representative immunoblots and semi-quantification analysis of renal α-SMA, VIM, and FN expression (n = 5–8 for α-SMA and VIM, n = 3–4 for FN). (E) qRT-PCR analysis of fibrosis markers *Acta2*, *Vim*, *Postn*, *Fn*, *Col1a1*, and *Col3a1* of mice kidney from corresponding group (n = 5–9). Data are presented as mean ± SEM; two-way ANOVA followed by Tukey's multiple comparisons test was used to determine statistical significance; $^{*}p < 0.05$ and $^{***}p < 0.001$ compared with the TP53RK$^{fl/fl}$+Sham group; $^{\#}p < 0.05$, $^{\#\#}p < 0.01$ and $^{\#\#\#}p < 0.001$ compared with the TP53RK$^{fl/fl}$+UIR group.

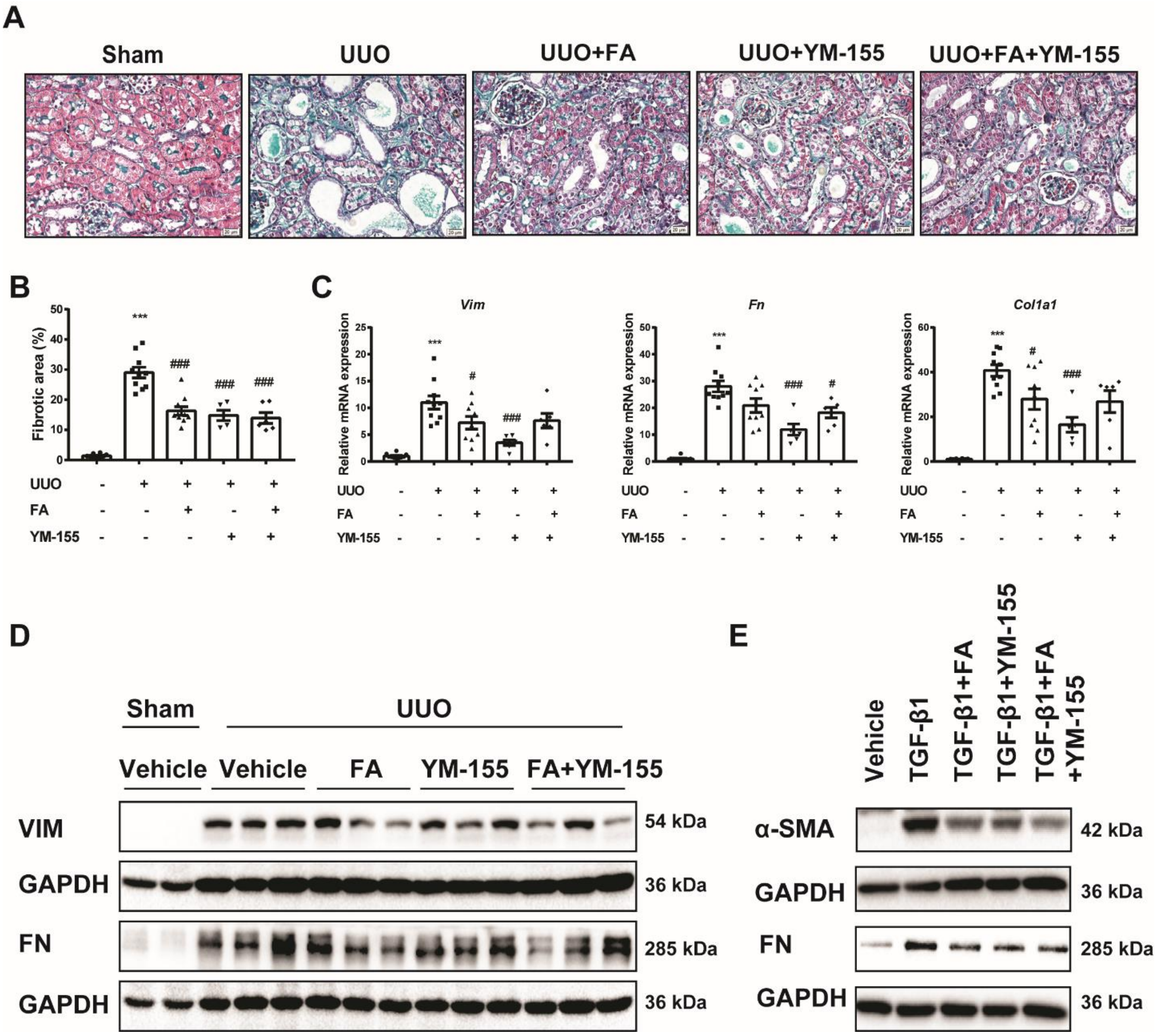

Figure S10 Pharmaceutical combination treatment of UUO mice with fusidic acid and YM-155

(A–B) FA was given to mice at 10 mg/kg/d and YM-155 was employed at 3 mg/kg/d, via intraperitoneal injection 24 h and 2 h before UUO surgery. Then the mice were treated daily for 7 consecutive days and sacrificed 2 h after the final injection. Deposition of total fibrosis in kidney tissues was determined by Masson's trichrome staining (A). Scale bar, 20 μm. Quantification analysis of fibrotic area was shown in parallel (B) (n = 6–10). (C) mRNA levels of *Vim*, *Fn*, and *Col1a1* in kidney tissues of corresponding groups (n = 6–10). (D) Representative immunoblots of VIM and FN in each group. (E) NRK-49Fs were pre-treated with FA (10 μM), YM-155 (2.5 nM) or both of the two inhibitors for 2 h and then stimulated with TGF-β1 (5 ng/mL) for 24 h. Protein expression of α-SMA and FN of each group was estimated. Data are presented as mean ± SEM; one-way ANOVA followed by Dunnett's multiple

comparisons test was used to determine statistical significance; $^{***}p < 0.001$ compared with the sham group; $^{\#}p < 0.05$, and $^{\#\#\#}p < 0.001$ compared with the UUO group.

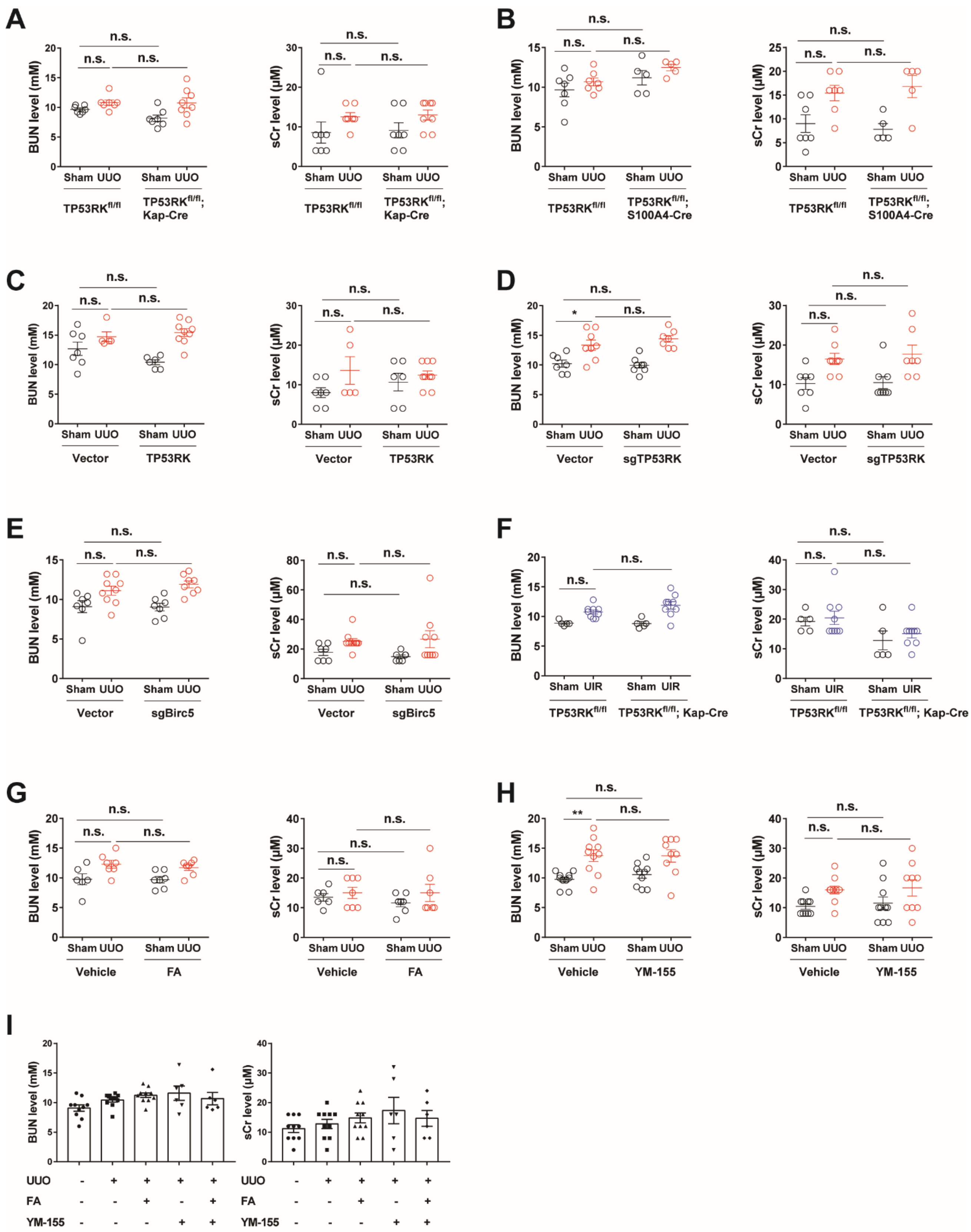

Figure S11 BUN and sCr levels of UUO and UIR mice

(A) TP53RK$^{fl/fl}$ and TP53RK$^{fl/fl}$; Kap-Cre mice were subjected to sham or UUO operation and euthanized at day 7.

BUN and sCr levels of each group were evaluated (n = 7–8). (B) $TP53RK^{fl/fl}$ and $TP53RK^{fl/fl}$; S100A4-Cre mice were subjected to sham or UUO operation and euthanized at day 7. BUN and sCr levels of each group were evaluated (n = 5–7). (C) TP53RK was overexpressed by hydrodynamic-based tail vein plasmid delivery. Mice were subjected to sham or UUO surgery 36 h later and euthanized 7 days after establishment of the model. Measurement of BUN and sCr in corresponding groups were shown(n = 5–9). (D) Thirty-six hours after hydrodynamic-based tail vein delivery of sgTP53RK plasmid, mice were subjected to sham or UUO surgery and euthanized 7 days after establishment of the model. BUN and sCr in corresponding groups were estimated (n = 7–8). (E) Thirty-six hours after hydrodynamic-based tail vein delivery of sgBirc5 plasmid, mice were subjected to sham or UUO surgery and euthanized 7 days thereafter. BUN and sCr in corresponding groups were estimated (n = 7–9). (F) $TP53RK^{fl/fl}$ and $TP53RK^{fl/fl}$; Kap-Cre mice were subjected to 45-min UIR and euthanized at day 21. Levels of BUN and sCr in corresponding groups were shown (n = 5–9). (G) Mice were pre-treated with fusidic acid (FA, 16 mg/kg/d) and subjected to sham or UUO operation and euthanized 7 days thereafter. Levels of BUN and sCr in corresponding groups were shown (n = 6–7). (H) Mice were pre-treated with YM-155 (3 mg/kg/d) and subjected to sham or UUO operation and euthanized 14 days thereafter. Levels of BUN and sCr in corresponding groups were shown (n = 9–10 in each group). (I) Mice were pre-treated with FA (10 mg/kg/d), YM-155 (3 mg/kg/d) or both of the two agents via intraperitoneal injection 24 h and 2 h before UUO surgery. Then the mice were treated daily for 7 consecutive days and sacrificed 2 h after the final injection. BUN and sCr levels of each group were evaluated (n = 6–10). Data are presented as mean ± SEM; two-way ANOVA followed by Tukey's multiple comparisons test was used to determine statistical significance in Figure A–H and the one-way ANOVA followed by Dunnett's multiple comparisons test was used to determine statistical significance in Figure I; $^{*}p < 0.05$ and $^{**}p < 0.01$ compared with the Vector+Sham or Vehicle+Sham group; n.s. represents no significant difference.

Table S1 Clinical parameters of CKD patients

Number	Gender	Age (yr)	Diagnosis	Interstitial fibrosis score	eGFR mL/(1.73m^2·min)	CKD stage	Treatment applied
1	Male	10	IgAN	6	60.27	Stage 2	None
2	Male	13	IgAN	9	34.21	Stage 3	Prednisone
3	Female	12	IgAN	3	143.23	Stage 1	Amoxicillin and Clavulanate Potassium
4	Male	13	IgAN	3	133.30	Stage 1	None
5	Female	6	IgAN	4	58.70	Stage 3	None
6	Female	14	LN-V	8	124.07	Stage 1	Prednisone
7	Male	1	Glomerulonephritis	7	43.02	Stage 3	None
8	Male	8	Thrombotic microangiopathy	9	19.08	Stage 4	Hemodialysis

9	Female	10	ANCA-associated vasculitis	9	15.44	Stage 4	None
10	Female	1	Thrombotic microangiopathy	5	26.49	Stage 4	Hemodialysis
11	Female	12	ANCA-associated vasculitis	8	15.01	Stage 4	CRRT, hemodialysis
12	Female	2	FSGS	5	69.37	Stage 2	None
13	Male	13	HSPN III-a	3	171.48	Stage 1	Methylprednisolone
14	Female	12	HSPN III-a	1	91.84	Stage 1	Dexamethasone, prednisone
15	Male	8	Subacute tubulointerstitial nephropathy	6	13.21	Stage 5	Hemodialysis, methylprednisolone
16	Female	9	HSPN III-b	1	108.78	Stage 1	None
17	Female	5	Glomerulonephritis	5	48.97	Stage 3	Hemodialysis, peritoneal dialysis

18	Female	11	FSGS	6	74.00	Stage 2	None

Abbreviations: CKD, chronic kidney disease; CRRT, continuous renal replacement therapy; eGFR, estimated glomerular filtration rate, FSGS, focal segmental glomerular sclerosis; HSPN, Henoch-Schonleinpurpuranephritis; IgAN, IgA nephropathy; LN, lupus nephritis.
